# Wrangling environmental exposure data: guidance for getting the best information from your laboratory measurements

**DOI:** 10.1186/s12940-019-0537-8

**Published:** 2019-11-21

**Authors:** Julia O. Udesky, Robin E. Dodson, Laura J. Perovich, Ruthann A. Rudel

**Affiliations:** 1 Silent Spring Institute, 320 Nevada Street, Newton, MA 02460 USA; 2 MIT Media Lab, 75 Amherst St, Cambridge, MA 02139 USA

**Keywords:** Exposure science, Environmental epidemiology, Environmental chemicals, Environmental monitoring, Quality assurance/quality control (QA/QC), Data validation, Exposure measurement, Measurement error

## Abstract

**Background:**

Environmental health and exposure researchers can improve the quality and interpretation of their chemical measurement data, avoid spurious results, and improve analytical protocols for new chemicals by closely examining lab and field quality control (QC) data. Reporting QC data along with chemical measurements in biological and environmental samples allows readers to evaluate data quality and appropriate uses of the data (e.g., for comparison to other exposure studies, association with health outcomes, use in regulatory decision-making). However many studies do not adequately describe or interpret QC assessments in publications, leaving readers uncertain about the level of confidence in the reported data. One potential barrier to both QC implementation and reporting is that guidance on how to integrate and interpret QC assessments is often fragmented and difficult to find, with no centralized repository or summary. In addition, existing documents are typically written for regulatory scientists rather than environmental health researchers, who may have little or no experience in analytical chemistry.

**Objectives:**

We discuss approaches for implementing quality assurance/quality control (QA/QC) in environmental exposure measurement projects and describe our process for interpreting QC results and drawing conclusions about data validity.

**Discussion:**

Our methods build upon existing guidance and years of practical experience collecting exposure data and analyzing it in collaboration with contract and university laboratories, as well as the Centers for Disease Control and Prevention. With real examples from our data, we demonstrate problems that would not have come to light had we not engaged with our QC data and incorporated field QC samples in our study design. Our approach focuses on descriptive analyses and data visualizations that have been compatible with diverse exposure studies with sample sizes ranging from tens to hundreds of samples. Future work could incorporate additional statistically grounded methods for larger datasets with more QC samples.

**Conclusions:**

This guidance, along with example table shells, graphics, and some sample R code, provides a useful set of tools for getting the best information from valuable environmental exposure datasets and enabling valid comparison and synthesis of exposure data across studies.

## Background

Chemical measurements play a critical role in the study of links between the environment and health, yet many researchers in this field receive little if any training in analytical chemistry. The growing interest in measuring and evaluating health effects of co-exposure to a multitude of chemicals [1, 2] makes this gap in training increasingly problematic, as the task at hand becomes ever-more complicated (i.e., analyzing for more and for new chemicals of concern). If steps are not taken throughout sample collection and analysis to minimize and characterize likely sources of measurement error, the impact on the interpretation of these valuable measurements can vary along the spectrum from false negative to false positive, as we will illustrate with real examples from our own data.

Some important considerations when measuring and interpreting environmental chemical exposures have been discussed in other peer-reviewed articles or official guidance documents. For example, a recent document from the Environmental Protection Agency (EPA) provides citizen scientists with guidance on how to develop a field measurement program, including planning for the collection of quality control (QC) samples [3]. The Centers for Disease Control and Prevention (CDC) also gives guidance related to collection, storage and shipment of biological samples for analysis of environmental chemicals or nutritional factors [4]. To assess the quality of already-collected data, LaKind et al. (2014) developed a tool to evaluate epidemiologic studies that use biomonitoring data on short-lived chemicals, with a focus on critical elements of study design such as choice of analytical and sampling methods [5]. The tool was recently incorporated into “ExpoQual,” a framework for assessing suitability of both measured and modeled exposure data for a given use (“fit-for-purpose”) [6]. Other useful guidance has been published, for example on automated quality assurance/quality control (QA/QC) processes for sensors collecting continuous streams of environmental data [7] and for establishing an overall data management plan, including documentation of metadata and strategies for data storage [8].

Despite these helpful documents, there is still a lack of readily accessible, practical guidance on how to interpret and use the results of both field and laboratory QC checks to qualify exposure datasets (i.e., flag results for certain compounds or certain samples that are imprecise, estimated, or potentially over- or under-reported) and this gap is reflected in the environmental health literature. While the vast majority of environmental health studies report robust findings based on high quality measurements, questions about measure validity have led to confusion and lack of confidence in some topic areas. For example, a number of studies have measured rapidly metabolized chemicals such as phthalates and bisphenol A (BPA) in blood or other non-urine matrices, despite the fact that urine is the preferred matrix for these chemicals. Phthalates and BPA are present at higher levels in urine and, when the proper metabolites are measured, there is less concern about contamination from external sources, including contamination from plastics during specimen collection [9].

More commonly, however, exposure studies simply do not adequately report on QA/QC or describe how QC results informed reporting and interpretation of the data. In the context of systematic review and weight of evidence approaches, not reporting on QA/QC may result in a study being given less weight. For example, the risk of bias tool employed in case studies of the Navigation Guide for Systematic Review includes reporting of certain QA/QC results in its criteria for a “low risk of bias” rating (e.g., reference [10]). When we applied the Navigation Guide QA/QC criterion to 30 studies of biological or environmental measurements that we included in a recent review of environmental exposures and breast cancer [11], we found that more than half either did not report QA/QC details that were required for a “low risk of bias” assessment, or if they did report QA/QC, did not interpret or use them adequately to inform the analysis (e.g., reported poor precision but did not discuss how/whether this could affect findings) (see Additional file 1 for details). Similarly, when LaKind et al. applied their study quality assessment tool to epidemiologic literature on BPA and neurodevelopmental and respiratory health, they found that QA/QC issues related to contamination and analyte stability were not well-reported [12]. Of note, several of the studies in our breast cancer review that did not provide adequate QA/QC information had their samples analyzed at the CDC Environmental Health Laboratory. It is helpful to include summaries of QA/QC assessments in published work even if researchers are using a well-established lab, because this provides a useful standard for comparing QA/QC in other studies.

Over many years of collecting and interpreting environmental exposure data, we have developed a standard approach for (1) using field and laboratory QA/QC to validate and qualify chemical measurement data for environmental samples and (2) presenting our QC findings in our research publications (e.g., reference [13]). These methods are based on data validation procedures from the EPA, Army Corps of Engineers, and U.S. Geological Survey [14–17] and the guidance of the many experienced chemists with whom we have collaborated. In this commentary, we compile our methods into a practical guide, focusing on how to use the information to make decisions about data usability and how to make the information transparent in publications. Our guide is organized in three sections presenting questions to consider during study design, implementation, and data analysis. We describe key elements of QA/QC, including for assessing precision, accuracy, and sample contamination, and we include suggested graphics (Additional files 2 and 4), and table shells (Additional file 2) that clearly present QC data, emphasizing how it may affect interpretation of study measurements. Minimizing and characterizing potential errors requires close collaboration between the researchers who may have designed the study and plan to analyze the data and the chemists performing the analysis, so our guidance also includes example correspondence (Additional file 2) to help establish this relationship at the start of a project.

We present a detailed approach based on our own studies, acknowledging that this is an example, not a one-size-fits-all approach. Every study is unique and some will require specialized quality assessment not covered here. Still, we anticipate that many environmental health scientists will find this example to be a useful framework for building their own processes.

### Wrangling guide

Our guide is organized by a series of questions that we ask when we start a new study and then again when we receive measurement data from the lab. Key QA/QC concepts are introduced in the Study Design section and are most thoroughly addressed in sections about Study Implementation and Data Interpretation.

Not every question is relevant to every study; for example, researchers working with a lab to develop a new analytical method will need to focus more on method validation and quality control than those using a well-established method and credentialed lab. Still, controlling for issues related to sample collection and transport remain important in the latter scenario, as does variation in method performance and/or sources of contamination when samples are analyzed at the laboratory in multiple batches. Our guidance is most relevant to targeted organic chemical analyses, which use liquid or gas chromatography, often in combination with mass spectrometry, to determine whether a pre-defined set of chemicals are present in samples. QA/QC approaches for non-targeted methods, where tentative identities are established by matching to a library of mass spectra such as the National Institute of Standards and Technology (NIST) database [18], are addressed elsewhere [19].

This guide is not a set of rules, but rather establishes a framework for evaluating and reporting QC data for chemical measurements in environmental or biological samples. While it may be most useful to environmental health scientists who have little or no experience in analytical chemistry, we hope that researchers with a range of experience will find it helpful to consult our approach for evaluating and presenting QC data in publications.

Because the number of QC samples available is often limited by budgetary constraints, many of the methods we use rely on visualization and conservative action (i.e., removing chemicals from our dataset or qualifying their interpretation unless there is evidence that the analytical method was accurate and precise) rather than on statistical methods. Whether statistical methods are incorporated or not, tabulating, visualizing, and communicating about QA/QC for environmental exposure measurements is important in order to reveal systematic error in the laboratory [20] or in the field and support future use of the data [6].

### Study design

#### What can we measure and how?

One of our first priorities when designing a new study is to consult with a chemist to establish an analyte list and method for analysis.

##### Chemical identities

Given the complexity of chemical synonyms, it is helpful to be as specific as possible when communicating about the chemicals to be analyzed. One approach is to send the lab a list of the chemical names (avoiding the use of trade names, which can be imprecise), Chemical Abstracts Service (CAS) numbers, and configurations (e.g., branched or linear, if relevant) of all desired analytes (see Additional file 1 for example correspondence). For biomonitoring, it is also important to determine if the parent chemical or metabolites will be targeted.

##### Matrix

Another consideration in developing the analyte list is what type of samples are available (if working with stored samples) or will be collected. As discussed previously, certain biological matrices are preferred over others for measurement, depending on the chemicals (e.g., reference [9]). Matrix type is also relevant for environmental samples; for example, physical chemical properties like the octanol air partitioning coefficient inform whether an analyte is more likely to be found in air or dust [21].

##### Method

The process of determining a final list of analytes will differ depending on whether the lab has an established method or is developing a new method, and whether it is targeted to a few chemicals with similar structure versus many chemicals with different properties (different polarities, solubilities, etc.). Targeting a broad suite of chemicals may limit the degree of precision and accuracy that can be achieved for each individual chemical, and the lab may need to invest substantial effort to develop a multi-residue method – that is, a method that can analyze for many chemicals at once – and determine a final list of target chemicals with acceptable method performance. In any case, a new method should be validated to characterize performance measures – precision, accuracy, expected quantitation and method detection limits, and the range of concentrations that can be quantitated with demonstrated precision and accuracy – before analyzing study samples. If the lab already has an established method for the chemicals of interest, the research team should review method performance measures to ensure they are consistent with study objectives.

##### Method – quantification

The method of quantification affects the types of QC data that are expected from the lab. Three common approaches include external calibration, internal calibration and isotope dilution (a form of internal calibration). External calibration, where the response (i.e., chromatogram peak) from the sample is compared to the response from calibration standards containing known amounts of the analytes of interest, is a simple method that can be used for a variety of different analyses. However results can be influenced by interference from other chemicals present in the sample matrix and resulting fluctuations in the analytical instrument response [22]. With internal calibration, on the other hand, one or more labeled compounds – either one of the targeted analytes or a closely related compound – are added to each of the samples just before they are injected into the instrument for analysis and used to correct for variation in the instrument response. The internal standard must be similar to the target compounds in physical chemical properties (e.g., a labeled polychlorinated biphenyl should not be used to represent a brominated diphenyl ether). Finally, for isotope dilution methods — which are the most accurate — labeled isotopes for each of the target compounds are added to samples prior to extraction. Additional internal standards are added to the samples just prior to injection to monitor loss of the labeled isotopes, and the analytical software then corrects for loss during sample extraction and for effects of the sample matrix (e.g., presence of other compounds in the sample that interfere with the analysis) [22]. Many laboratories that analyze chemical levels in blood, urine, or tissues — including the CDC National Exposure Research Laboratory — use isotope dilution quantification. However isotopically labeled standards are not available for every compound and may be cost prohibitive. If quantification is by internal or external calibration, researchers will likely need to review and report more extensive QC data from the lab compared to when using isotope dilution, as discussed in Study Implementation: What QA/QC is needed?

##### Method – sensitivity

Another important factor in selecting a method is to make sure it is sensitive enough to detect the anticipated concentrations in the field samples (samples submitted to the lab) down to levels that are relevant to the research question. For example, commercial labs measuring environmental chemicals may establish reporting limits to meet the needs of occupational or regulatory safety compliance testing; these limits may be much higher than levels that are meaningful for research questions about general population exposure and could result in most data being reported as non-detect or qualified as estimated and imprecise. On the other hand, lower reporting limits generally translate to more expensive testing, so researchers have the opportunity to balance sensitivity and cost.

#### How to minimize sample contamination?

There are ample opportunities for sample contamination during collection, storage, shipment and analysis, especially when targeting ubiquitous chemicals commonly encountered in consumer products and in home and office furnishings or laboratory equipment. An important aspect of method validation is to check for contamination of samples during field activities, from collection containers, during transport and storage, and during laboratory extraction and analysis (see discussion of blanks in the Study Implementation section). The CDC’s guidance on sample collection and management identifies some possible sources of contamination when analyzing for common chemicals like plastics chemicals, antimicrobials and preservatives in blood or urine. Key considerations, depending on the particular chemicals being targeted, include selecting appropriate collection containers (e.g. glass containers if analyzing for plastics chemicals), avoiding the use of urine preservatives (e.g., when analyzing for parabens, BPA), and providing adequate instructions to participants collecting their own samples (e.g., avoid using antimicrobial soaps or wipes during collection) [4]. As noted previously, contamination can also be minimized in biomonitoring of some chemicals by measuring a metabolite rather than parent chemical, and possibly by measuring a conjugated rather than free form of the metabolite [9]. In some cases, the lab may need to pre-screen collection containers or other sampling materials to see if they contain any target chemicals. For example, when we used polyurethane foam (PUF) sorbent to collect air samples for analysis of flame retardants, plastics chemicals and preservatives, we asked the lab to pre-screen the PUF matrix for target analytes. Another important precaution was to ship the samplers wrapped in aluminum foil that had been baked in a muffle furnace to ensure it was clean and uncoated.

#### How will the lab report the data?

Three key elements of data typically reported by the lab are the identity of the chemical, the reporting limit for each chemical and sample, and how much of each chemical is present in each sample. Sometimes an additional measure is needed to normalize mass of chemical per sample, for example, grams of urinary creatinine, urine specific gravity, grams of serum lipid, or cubic meters of air (see reference [5] for discussion of issues related to matrix adjustment and presentation of measurements).

##### Chemical identities

It is helpful to request in advance that the lab report CAS numbers and configurations (if relevant) along with chemical names (see Additional files 2 and 3 for example reporting requests).

##### Reporting limits

Common terms used by laboratories to discuss reporting limits include instrument detection limit (IDL), method detection limit (MDL) and limit of quantitation (LOQ). The IDL and MDL are both related to the level of an analyte that can be detected with confidence that it is truly present. The IDL captures the smallest true signal (change in instrument response when an analyte is present) that can be distinguished from background noise (variation in the instrument response to blank samples), while the MDL takes into account additional sources of error introduced during sample preparation (e.g., the extraction process, possible concentration or dilution of samples) and thus is higher than the IDL. The MDL is also often referred to as the limit of detection (LOD) or detection limit (DL). The LOQ, on the other hand, describes the lowest mass or concentration that can be detected with confidence in the *amount* detected. The reporting limit (RL) or method reporting limit (MRL), which is either the lowest value that the lab will report or the lowest value that the lab will report without flagging the data as estimated, is often (but not always) the same as the quantitation limit or LOQ.

Before submitting samples for analysis, it is helpful to find out (1) the methods and terminology that the laboratory will use to describe reporting limits (LOD, LOQ, etc.) and (2) whether reporting limits will be consistent within a chemical or whether limits could vary between samples or batches. Equally critical is to clarify how the lab will report non-detects. Several different values could appear in the amount or concentration fields for non-detects, including but not limited to zeroes, the detection limit, the reporting limit or “ND.”

##### Amount

Another important point to discuss in advance with the laboratory is how they will report values for compounds with a confirmed identity but measured at levels below what can be accurately quantitated. For example, when measuring chemicals of emerging interest, we ask laboratories to report estimated values below the RL and we flag them during data analysis. This practice has some limitations [23] but is preferable to falsely reducing variance in the dataset by treating estimated values below the RL as equivalent to non-detects below the detection limit. Non-detects can present significant data analysis challenges, and while a discussion of the best available methods and the problems with common approaches such as substituting the RL, RL/2 or zero for non-detects is beyond the scope of this commentary, it is a critical issue and we refer the reader to several helpful resources [23–26]. Reporting estimated values is not standard practice for many laboratories, so it is important to raise this issue early on (see Additional file 2 for example correspondence). If the lab reports data qualifier flags, it may be necessary to clarify the interpretation of those flags, including but not limited to which flags distinguish non-detects from detects above the MRL and estimated values. It is best not to make assumptions.

### Study implementation

#### What QA/QC is needed?

QA/QC occurs both inside and outside the analytical laboratory (see Table 1). Field QC samples, namely blanks and duplicates, capture the sum of contamination and measurement error from collection, storage, transport, and laboratory sources. We base the number of QC samples we collect in the field on budget and our sample size, generally aiming for at least 20% QC samples (e.g., if collecting 80 field samples then collect 16 field QC samples), though a higher percentage is needed in small studies. Lab analysts should be blinded to the identity of field QC samples whenever possible. Maintaining blinding can be challenging, so it is worth putting some thought into sample names (e.g., QC samples should not have obviously different IDs than other samples, should not be labeled with a “D” for duplicate or “B” for blank). Logs retained at the site must contain sufficient information to allow the data analysts to identify field QC samples and sample types.

**Table 1:** Summary of QC sample types, interpretation, and possible actions

QA/QC Concept	Measure	Interpretation	Possible Actions
*Accuracy*	Lab control sample recoveries and/or matrix spike recoveries Certified reference material Isotope dilution quantification	Measure of whether the analytical method produces accurate quantification for each compound. Matrix spike recovery evaluates matrix effects on accuracy, such as interferences. Isotope dilution is the most rigorous approach to generating accurate measurements in biomonitoring.	• Drop compounds with inaccurate quantification from the data analysis, discuss with lab whether improvements can be made for future analyses. • If problems are modest and batch-specific, include batch as a covariate in regression model.
*Extraction efficiency*	Surrogate spike recovery in each sample	Measure – for each field sample - of whether the chemical is extracted completely from the sample matrix, (e.g., blood, dust). Isotope dilution approaches capture and correct for differences in extraction efficiency.	• Consider dropping samples with poor surrogate recovery from data analysis. • Consider applying a surrogate correction factor (1/fraction recovery) if the recovery is consistent (± 15–20% in standard deviation).
*Detection limit*		Level above which the lab can detect with confidence that the analyte is present in the sample. Common terms include: Instrument detection limit (IDL), Detection limit (DL), Method detection limit (MDL), Limit of detection (LOD)	• See Method Reporting Limit.
*Quantitation limit*		Level above which the lab can quantify with confidence the amount of chemical in the sample. Common terms include: Practical quantitation limit (PQL), Limit of quantitation (LOQ), Laboratory quantitation level (LQL), Contract required quantitation limit (CRQL)	• See Method Reporting Limit.
*Method Reporting Limit (MRL)*	Levels detected in blanks (lab-blind field blanks, solvent blanks, matrix blanks, storage blanks, other types)	Level above which the researcher is confident that the reported chemical measurement reflects a signal from the media sampled, considering all sources of measurement error, especially potential contamination during sample collection and handling as well as in the laboratory.	• Determine MRL by comparing the lab limit (quantitation limit, unless not reported, in which case detection limit) to the levels in the blanks for each compound. • Qualify reported values below the MRL as “estimated”.
*Potential contamination / Analytical bias*	Levels detected in blanks (lab-blind field blanks, solvent blanks, matrix blanks, storage blanks, other types)	Measure of confidence in accuracy of values reported above the MRL.	• If evidence of contamination, consider dropping a compound or dropping results for a compound in a particular batch. • Identify source of contamination (e.g., lab vs field equipment) to inform future work.
			• For compounds with consistent contamination in blanks, researchers may correct field sample quantity by subtracting the amount attributed to contamination. This is most important when contamination is significant relative to sample values (e.g., > 10%) and for comparisons with external data.
*Precision*	Relative percent difference (RPD) for side-by-side duplicate samples (lab-blind) or split samples (lab-blind if possible)	A measure of reproducibility of field measurements, including analytical variability and sampling variability.	• Flag compounds with > 30% RPD. • Consider precision in combination with other QA/QC when deciding to qualify results.

QC samples prepared in the lab can include spiked samples or certified reference materials (CRMs) for target chemicals to evaluate the accuracy of the analytical method, surrogate compounds added to field samples to estimate recovery during extraction and analysis, and blanks to assess contamination with target chemicals from some source in the laboratory. While laboratories generally conduct rigorous review of their own QC data, considering lab and field QC together can help to identify specific sources of contamination, imprecision, and systematic error, so we typically request to review the lab’s raw QC data in conjunction with the field QC data.

##### Spiked samples and certified reference material

Spiked samples and CRMs establish the accuracy of the method by assessing the recoveries of known amounts of each target chemical from a clean or representative matrix. A CRM is a matrix comparable to that used for sampling (e.g., drinking water) that has been certified to contain a specific amount of analyte with a well-characterized uncertainty. If CRMs aren’t available, the laboratory can prepare laboratory control samples (LCSs) by spiking known amounts of target chemicals into a clean sample of the matrix of interest, such as a dust wipe, air sampler, purified water or synthetic urine or blood that has been analyzed and shown to be free of the analytes of interest, or to contain a consistent amount of analytes of interest that can be subtracted from the amounts measured in the spiked sample to calculate a percent recovery. The LCS or CRM – at least 1 per analytical batch – is run through the same sample preparation, extraction, and analysis as the field samples to capture the accuracy of the complete method; calculating the percent of the known/spiked amount recovered for each analyte tells us whether the method is accurate in the matrix.

Another type of spiked sample, called a matrix spike, can be used to check the extraction efficiency for a complex sampling matrix that may interfere with the analysis. These samples are typically included if there is concern about interference from the sampling matrix, for example, with house dust, soil or sediment samples, consumer products, or biological samples like blood. Instead of recovery from a clean matrix, these QC checks capture recovery from a representative field sample. Here the “matrix” refers to all elements of the sample other than the targeted analytes; this includes the sampling medium (e.g., dust, PUF, foam) itself as well as any other chemicals present in the sample that might interfere with measurement of target chemicals. A matrix spike can be created, for example, by splitting a representative sample collected in the field and spiking the target analytes into one half prior to extraction and analysis. The recovery of spiked analyte is determined as the amount measured in the spiked sample minus the amount measured in the non-spiked sample divided by the spike amount. A limitation of this approach is that the analytes are spiked in an already dissolved state, so it is possible that the analytes in the environmental matrix would not be extracted as readily from the matrix as the spiked chemicals. Thus, the true extraction efficiency may be lower than represented by the matrix spike.

For newly developed methods where performance is not characterized, we request results for all recoveries of spiked samples and/or CRMs so that we can perform visual checks that have at times revealed systematic problems with the analytical method that were not noted by the lab (see Data Interpretation: Is the method accurate? for discussion). For well-established methods, and particularly when isotope dilution quantification is used, it is sufficient to request a table summarizing the spike recovery or CRM recovery results (by batch, if relevant) for reporting in publications.

##### Surrogate recovery standards

Whereas recoveries from LCSs, matrix spikes and/or CRMs tell us about the performance of the method in a clean or representative matrix, surrogate compounds are used to evaluate recoveries from individual samples. Recoveries of surrogate compounds can help identify any individual samples that may have inaccurate quantification, for example due to extraction errors or chemical interferences. Surrogates, like internal standards, are spiked into each sample, however surrogates are added prior to sample extraction to assess the efficiency of this process. Internal standards, on the other hand, are added after extraction, just prior to injection into the chromatographic system, to account for matrix effects and other variation in the instrument response during analysis. The ideal surrogate is a chemical that is not typically present in the environment but that is representative of the physical and chemical properties of target analytes [16]. It is best to have a representative surrogate for each individual chemical, though when analyzing for numerous chemicals at once with multi-residue methods, cost and time restraints may result in one or a few surrogates being selected to represent a class of compounds. In this case it is critical that the lab selects an appropriate surrogate.

For analyses using external or internal calibration, we ask the lab to provide us with the recovery results for each surrogate in each sample, so that we can flag any samples or compounds that might have had extraction problems. However if the lab uses isotope dilution quantification, we are less concerned about obtaining this raw data from the laboratory given that the reported results are already automatically corrected for extraction and matrix effects.

##### Blanks

Collecting and preparing several types of blank samples helps us to distinguish sources of contamination. Laboratory blanks alert us to possible contamination originating in the lab. These blanks can capture contamination during sample extraction (solvent blanks), from reagents and other materials used in the analytical method (solvent method blanks) or from “typical” background levels of target analytes present in the sampling matrix (matrix blanks). Field blanks, on the other hand, capture *all* possible contamination during sample collection and analysis. Field blanks are clean samples (e.g., distilled water, air sampling cartridge detached from pump immediately following calibration) that are transported to the sampling location and exposed to all of the same conditions as the real samples (e.g., the sampler is opened, if applicable) except the actual collection process. We aim for at least 10% of our samples to be field blanks, with an absolute minimum of 3 field blanks.

Unfortunately, in some cases there aren’t good options for representative field blanks. For example field blanks can be created for biomonitoring programs by taking empty collection containers into the field and using purified water or synthetic urine or blood to create a blank [4]. However, important short-comings of this approach are that (1) it is difficult to capture contamination that can be introduced by sample collection materials such as needles and plastic tubing used to collect blood, (2) water may not perform the same as urine or blood in the extraction and analysis, and (3) the lab will likely be able to identify the field blanks. Similarly, it is difficult to maintain lab blinding when using a “clean” matrix like vacuumed quartz sand as a field blank for vacuumed house dust.

##### Duplicates

Collecting side-by-side duplicate samples in the field helps assess the precision of both the sample collection and analytical methods. Duplicate samples can also be created by collecting a single sample and splitting it prior to analysis, which is the only option for biological samples; however, this method only captures the precision of the analysis process [14, 17] and could lead to un-blinding of the lab analyst, if for example the split samples are noticeably smaller than others. When planning for duplicate collection, the best practice is to label these samples so that the lab analyst is blinded to duplicate pairs (i.e., use different Sample IDs for the two samples). Ideally, researchers should plan to collect or create (that is, split) one duplicate pair per every 10–20 samples collected, and spread duplicate pairs across analytical batches.

##### Analytical batches

Analytical performance can shift over time and even between multiple extractions or instrument runs within a short time window. Laboratories often analyze samples in multiple batches, that is, sets of field samples and associated laboratory QC samples that are analyzed together in one analytical run. The time between batches can vary from days to months or even years, though ideally this time span is minimized in order to maintain consistent equipment and procedures throughout the study.

Two approaches help address batch-to-batch variability: (1) randomizing participant samples between batches by specifying the order and grouping of samples (and blind field QC samples) when submitting samples to the lab (this may require corresponding with the lab to determine the batch size in advance), and (2) running CRMs – such as standard reference material (SRM) from NIST [27] – in each batch of samples in order to characterize drift. When CRMs are not available, another option is for the researcher to prepare identical/split reference samples. We have done this, for example, by pooling together several urine specimens and making many aliquots of the pool, then including 1–2 blinded samples from this pool with each set of samples we send to the lab. If the laboratory analysis is performed in multiple batches, all QC elements should be examined on a batch-specific basis. Not every laboratory will specify whether or not samples were analyzed in batches; it is a good idea to request that a variable for batch be included in the results report.

In Additional files 2 and 3, we provide example correspondence for requesting QC data and consistent formatting from the lab.

### Data interpretation

#### What was measured?

##### Chemical identities

No amount of QA/QC can save a dataset from basic misunderstandings about what is being reported. After receiving data, it is helpful to ask the chemists to double check the analyte list (chemical name, CAS, isomer details) against the list of standards used in the analysis, particularly if this information was not included in the report from the lab. It is worthwhile to make this verification even when chemical identities were specified in advance of the analysis as it is possible that the standard used for analysis was slightly different than planned. Only through this process, for example, did we discover that a lab had accidentally purchased a standard for 2,2,4-trimethyl-1,3-pentanediol isobutyrate rather than 2,2,4-trimethyl-1,3-pentanediol diisobutyrate (two different chemicals).

Table 2 summarizes some steps for getting acquainted with a new dataset received from the lab. We have also published sample R code on GitHub that may be helpful for getting acquainted with a new dataset, including examining trends in QC and field samples over time [28].

**Table 2:** Get acquainted with your data

*1. Verify* chemical identities.	
○ Check CAS number, chemical name, isomer type of reported analytes vs. analytical standards purchased by lab (see Additional file 2 for example correspondence).	
*2. Count* (overall and by batch) the number of:	
○ “Real” samples – compare these to the chain of custody that lists the samples submitted for analysis to make sure all submitted samples were analyzed	
○ Lab control and/or matrix spike recoveries	
○ Reference samples (e.g., CRMs)	
○ Surrogate spike recoveries	
○ Blanks (solvent method, field, matrix, other)	
○ Duplicates	
*3. Examine* any data qualifier flags reported by the lab and make sure interpretation is clear.	

#### Were there trends over time?

##### Analytical batches

**Fig. 1:**
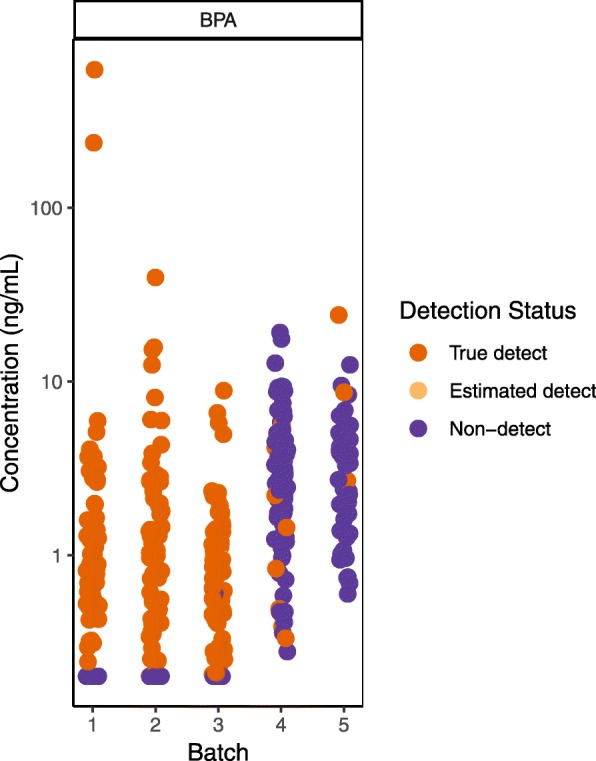
Visualizing urine sample results by analytical batch (data not yet published) revealed that sample-specific detection limits in later batches were higher and in the range of sample results in previous batches. After discussing with the laboratory, samples in later batches were re-analyzed to achieve lower detection limits

Examining results by batch or even by sample run order can reveal trends in QC samples over time, identifying systematic laboratory errors that may be missed by summary statistics or visualizations [20]. Shifts in method performance over time may require batch-specific corrections or dropping or flagging data from certain batches. Notably, a trend in QC sample results over time can be problematic even if they remain within the acceptable limits established by the lab. In our own work, for example, examining our data by analytical batch revealed an upward trend in sample-specific detection limits for some analytes, such that detection limits in later batches were within the range of sample results from earlier batches (Fig. 1). The detection limits in the later batches still met the specifications of our contract with the lab, but it was clear that we would not be able to compare results in the latter two batches to those in the first three. We showed the plot in Fig. 1 to the lab and they agreed to re-analyze the samples in the later batches, which resulted in more consistent detection limits.

#### Is the method accurate?

##### Spiked samples and certified reference material

Table 3 outlines our approach for analyzing LCS or matrix spike recovery or CRM data. The approach is similar for all of these samples. However one distinction is that if LCS recovery and other QC measures, such as lab blanks (matrix, solvent method, or other) are acceptable, a poor matrix spike recovery (higher or lower than acceptable bounds) can alert chemists to interferences from matrix effects, and suggest steps to address this such as matrix-matched calibration [17]. We typically only use data for analytes that have average LCS, matrix spike and/or CRM recoveries between 50 and 150%, though this decision criterion can be adjusted based on the needs of the project. If we do retain data for chemicals with spike or CRM recoveries outside of this acceptable range, we note in publications that concentrations in our data may be under- or over-reported.

**Table 3:** Spiked samples and certified reference material

Approach (see Additional file 4 for example of this approach with real data):	
*1. Summarize* percent recoveries for each chemical across analytical batches and flag those chemicals with average recoveries outside of a pre-established acceptable range.	
□ We typically apply an acceptable range of 50–150% recovery for most environmental samples, particularly when we are analyzing for new chemicals or combinations of chemicals for which methods are not well-established. For well-established methods, a more conservative range – 80-120% recovery – is appropriate.	
*2. Visualize* percent recoveries for each chemical across analytical batches to assess consistency.	
□ If recoveries for a particular chemical or chemicals are consistently out of range (> 150% or < 50%) across multiple batches, this should be discussed with the laboratory analyst.	
○ If the laboratory analyst agrees that the method was not successful, we drop the chemical(s) from our dataset. We do not report values or include such chemicals in any data analyses.	
○ If the laboratory analyst can explain the reason for consistent high or low recoveries and has confidence in the ranking and relative values of the reported sample data, the reported values can be used for many data analyses, but it will be difficult to compare with levels from another study.	
□ If recoveries from one or a few batches are out of range, we are concerned that results in those batches might be over/under-estimated compared to the rest. One way to investigate this concern is to look for corresponding systematic differences in sample data (see Additional file 4: Figure S3).	
○ If field samples have been randomized into batches, we check if the variation in sample results correlates with spiked sample or CRM recoveries by batch. Note: we still go through this step even if we were not able to randomize field samples, but in this case it can be very challenging to distinguish systematic analytical variation from other possible sources of variation in sample results between batches (e.g., if samples in different batches were also collected during different seasons).	
▪ If there are systematic differences (e.g., the sample results for a chemical are higher in the batch where the spike or CRM recovery was high, or if only one batch, the sample results for a chemical with high spike or CRM recovery are much higher than previously reported levels), we consider dropping the chemical results from the affected batches from the dataset. If an identical/split reference sample was analyzed in each batch, these results can also be helpful to resolve questions about whether and how to use the data in this case.	
▪ If there are no obvious systematic differences, we keep the chemical in our dataset, but flag the results for that chemical in the batch with the out-of-range spiked sample or CRM recovery.	
Reporting:	
□ We note in summary statistics when the average spiked sample or CRM recovery for a particular chemical was out of range.	
□ We note whether levels in our study might be systematically over- or under-reported (i.e., because of consistent high or low spiked sample or CRM recoveries). We especially note this if comparing to levels from another study.	
□ For chemicals with low/high recoveries in certain batches, we may perform sensitivity analyses – for example, by including lab batch as a covariate in regression analyses, though this can be challenging for small datasets.	

**Fig. 2:**
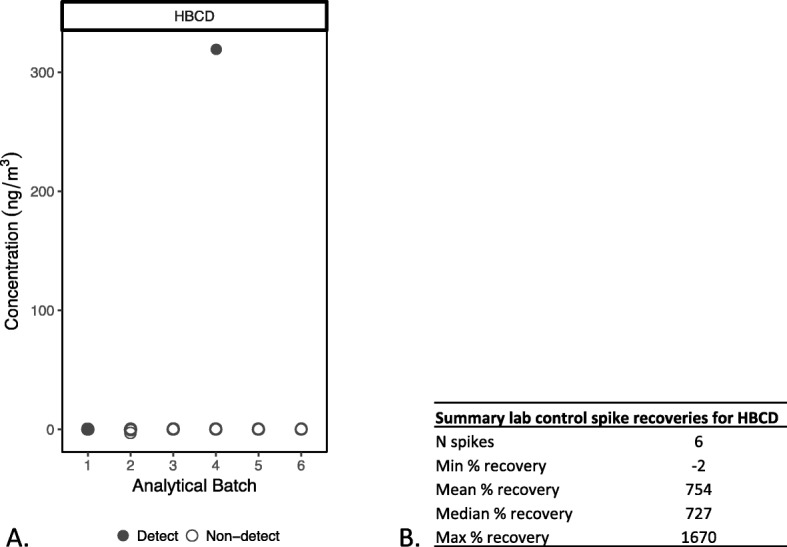
**a** Results for flame retardant HBCD measured in air samples collected in 105 homes. All but three samples were non-detects (open circles). Samples were analyzed in six different analytical batches. **b** Summary of laboratory control spike recovery data for HBCD across the six analytical batches shows very poor accuracy and indicates no confidence for this analyte in the indoor air samples

Figure 2 illustrates a case from our own data where the laboratory reported that 1,2,5,6,9,10-Hexabromocyclododecane (HBCD), a brominated flame retardant, was mostly “not detected,” but the LCS recoveries, which ranged from − 2 to 1670% and averaged about 750%, indicated that the method was not able to accurately quantify this chemical. We removed this compound from our dataset and did not report on it. Examining spike recoveries thus prevents us from reporting a chemical as “not detected,” or from reporting an unreliable detect, if the analytical method is not performing accurately for that compound.

A summary of the recovery information should be included in the peer-reviewed manuscript to demonstrate accuracy. See Additional file 2: Tables S1-S2 and Figure S1 for an example of how to present this information.

#### Were there problems with certain samples?

##### Surrogate recovery standards

When isotope dilution quantitation with automatic recovery correction is not employed, we review the surrogate recovery standard data for each individual sample, generally considering 50–150% recovery to be acceptable. Interpretation of an out-of-range surrogate recovery depends both on its direction and on the levels of the associated analytes (i.e., those represented by the surrogate compound) measured in the sample. In samples with low surrogate recoveries, the concern is that if similar target analytes are present in the sample, the measurements will be underestimated/biased low. For samples with high surrogate recoveries, on the other hand, we can be confident that similar target compounds should be detected if present, but the amount may be overestimated or biased high. If surrogate recoveries are out-of-range in all samples, and particularly if they are also out-of-range in blank samples, this is likely indicative of a broader problem with the analytical method [16, 29]. Table 4 outlines our approach for analyzing surrogate recovery data.

**Table 4:** Surrogates

Approach (see Additional file 4 for example of this approach with real data):	
*1. Count* high and low recoveries for each surrogate chemical across analytical batches.	
□ We typically apply an acceptable range of 50–150% recovery for most environmental samples, particularly when we are analyzing for new chemicals or combinations of chemicals for which methods are not well-established. For well-established methods, a more conservative range – 80-120% recovery – would be appropriate.	
*2. Identify* any sample where all surrogate recoveries were low (e.g., < 50%). This suggests a potential problem with the extraction for that sample.	
□ Discuss with lab analyst. Consider dropping sample.	
*3. Visualize* surrogate recoveries for *QC samples* (lab blanks, lab control or matrix spikes) across analytical batches. See Additional file 4: Figure S4 for an example.	
□ If these recoveries are out of range, this suggests a larger problem with the analytical method rather than with particular samples. Summarize information about the surrogate recoveries in the QC samples as well as lab control or matrix spike recoveries for the associated chemicals and discuss with lab analyst.	
*4. Visualize* percent recoveries *across all samples* for each surrogate, by analytical batch. See Additional file 4: Figure S6 for an example.	
□ Note any trends (upward or downward) in the distribution of surrogate recoveries across batches. Such trends should be discussed with the laboratory analyst, even if all recoveries are in the 50–150% acceptable range (see Fig. 3 for an example).	
□ If the surrogate is a deuterated version of one of the target chemicals, it can be helpful to compare a plot of the surrogate recoveries by batch to the sample data for the corresponding un-deuterated target chemical by batch. We would be concerned – and would seek guidance from the lab analyst – if we saw a trend for the target chemical results that matched the trend in the surrogate recoveries.	
□ Note if many surrogate recoveries (e.g., more than half) are out of range in a particular lab batch. If yes, flag the results in that batch for the chemical(s) represented by that surrogate.	
*5. Visualize* sample results flagged by surrogate recoveries. For each individual sample with an out-of-range recovery for a surrogate, flag the results for the chemical(s) associated with that surrogate. Plot all sample data with indicators (e.g., different colors) for whether the representative surrogate for each sample was out of range. See Additional file 4: Figure S7A-D for an example.	
□ Note whether samples with high surrogate recoveries consistently have the highest results for the associated chemical(s).	
○ If yes, we would be concerned that samples with high recoveries are all overestimated. Discuss with lab analyst. Consider applying a surrogate correction factor to sample results (multiplying by 1/fraction recovery).	
□ Note whether samples with low surrogate recoveries were consistently non-detects or very low-level detects for the associated chemical(s).	
○ If yes, we would be concerned that samples with low recoveries are all underestimated. Discuss with lab analyst. Note in publications that levels and detection frequencies for associated chemicals might be underestimated.	
Reporting:	
□ In summary statistics, we note whether any maximum value is from a sample associated with a high (> 150%) surrogate recovery and note that in this case the maximum might be overestimated. Similarly, if 100% of samples are detects, we also flag the minimum value if it is from a sample associated with a low (< 50%) recovery and note that in this case the minimum might be underestimated.	
□ For any statistical analyses, if possible (i.e., if large enough dataset) we run sensitivity analyses:	
○ Excluding samples with out-of-range surrogate recoveries.	
○ Controlling for lab batch, if surrogates were problematic for a particular batch.	

**Fig. 3:**
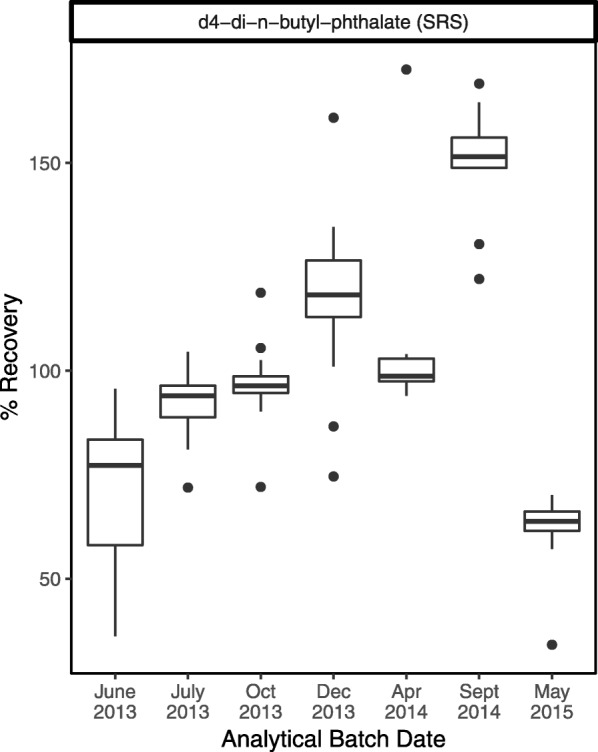
In this example from our data, recoveries of surrogate d4-di-n-butyl-phthalate from air samples showed notable upward and downward trends over time, despite largely staying within the 50–150% acceptable bounds. Here we were examining surrogate recoveries in batches of samples from different studies analyzed at the same laboratory. The last two batches (Sept 2014 and May 2015) were from the same study but collected approximately a year apart per the study design

Figure 3 shows an example where our examination of surrogate recoveries on a batch-specific basis indicated trends in the recoveries over time, even though most remained within the generally acceptable range (50–150%). This plot led to a discussion with the lab analyst, who suggested that stock solutions for surrogate compounds may have concentrated over time as solvent evaporated, until a new stock solution was prepared for the last batch. On the advice of the lab analyst, we looked at trends in the “spike check” – solvent that is spiked with target analytes but not extracted or concentrated – sample recoveries. Spike check recoveries indicated good reproducibility, giving us confidence that the drift in surrogate recoveries did not reflect changes in instrument calibration over time.

#### Is there evidence of contamination or analytical bias?

##### Blanks

Once we have determined that we can accurately measure the target analytes in our sampling matrix, the next step is to ensure that we are confident about whether those target analytes came from the study site or participant – or from somewhere else. Table 5 outlines our approach to reviewing data from blank samples. When it is not straight forward to collect field blanks (e.g., for blood samples), any assessment of contamination introduced from sampling (e.g., pre-screening of collection materials) should be thoroughly described and limitations acknowledged.

**Table 5:** Get acquainted with blanks

Approach (see Additional file 4 for example of this approach with real data):	
*1. Summarize* results across all chemicals by blank type (e.g., field blank, solvent method blank, matrix blank, etc.), with non-detects set to zero. For chemicals with no detects in blanks, the MRL will equal the lab reporting limit and none of the subsequent steps in Tables 6 or 7 are needed.	
For chemicals detected in blanks:	
*2. Visualize* levels in blanks by blank type. Set non-detects to ½ lab reporting limit and plot by analytical batch.	
□ Consider whether blank detects are consistent across batches. Note whether detects seem to occur mostly in one type of blank which could indicate a source of contamination in the lab or field.	
○ If a particular source is suspected, we investigate (talk to lab, look at field logs, etc.).	
*3. Visualize* levels in blanks by blank type along with field samples by analytical batch. Set non-detects to ½ lab reporting limit.	
□ Note whether blanks are in range of the samples.	
□ If field samples have been randomized into batches, check if variation in sample results correlates with blank results by batch. Note: we still go through this step even when we were not able to randomize field samples, but it is more challenging to distinguish whether contamination is driving differences in sample results in a particular batch or whether other explanations are more likely (e.g., all samples in one batch were collected in a different season or from a particular study site).	

**Fig. 4:**
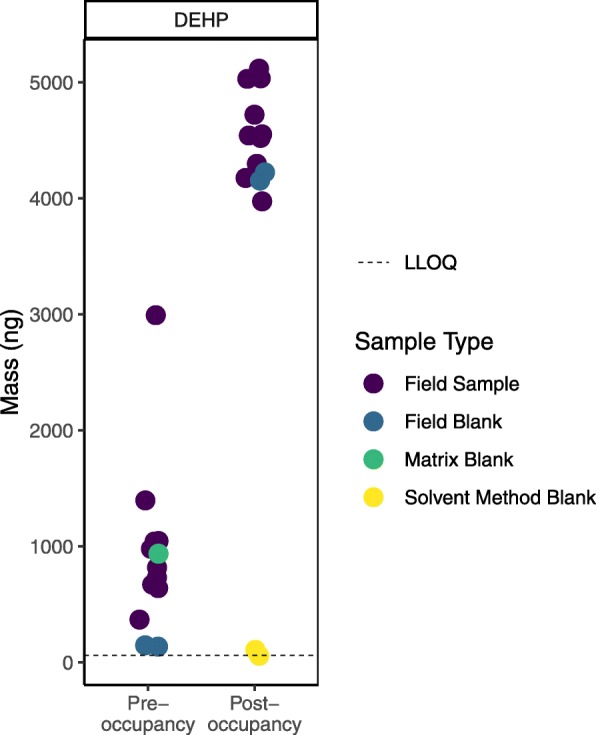
Phthalate DEHP measured in air in college dorm rooms before and after occupancy (data not yet published). Levels in our samples (purple dots) were higher post- compared to pre-occupancy, but this plot revealed that levels in field blanks (blue dots) were also higher post- compared to pre-occupancy and within the range of field samples. We also saw a matrix blank (green dot) well within the range of the field samples in the pre-occupancy batch. These data suggest DEHP contamination in both batches; for the post-occupancy batch, we hypothesized this might have come from the plastic bags in which the samplers were shipped. We will not report results for this chemical from this study, given the evidence of contamination. LLOQ = Lower Limit of Quantitation

Figure 4 illustrates an example from our study comparing levels of chemicals in air in college dorm rooms before and after students moved in (data not yet published) where field blanks proved particularly crucial. Our first look at the sample data suggested that bis(2-ethylhexyl) phthalate (DEHP), a chemical commonly used in plastics, was present at notably higher levels after students moved in. However, upon further review, we found that DEHP levels in the field blanks were also higher and in the range of the sample data at the post- compared to pre-occupancy time point. At the same time, levels of DEHP in the laboratory blanks (matrix and solvent method) were not elevated. A conversation with the lab revealed that different plastic bags may have been used to transport samples during the later round of sampling (i.e., the post-occupancy sampling). These bags may have contained higher levels of DEHP.

**Fig. 5:**
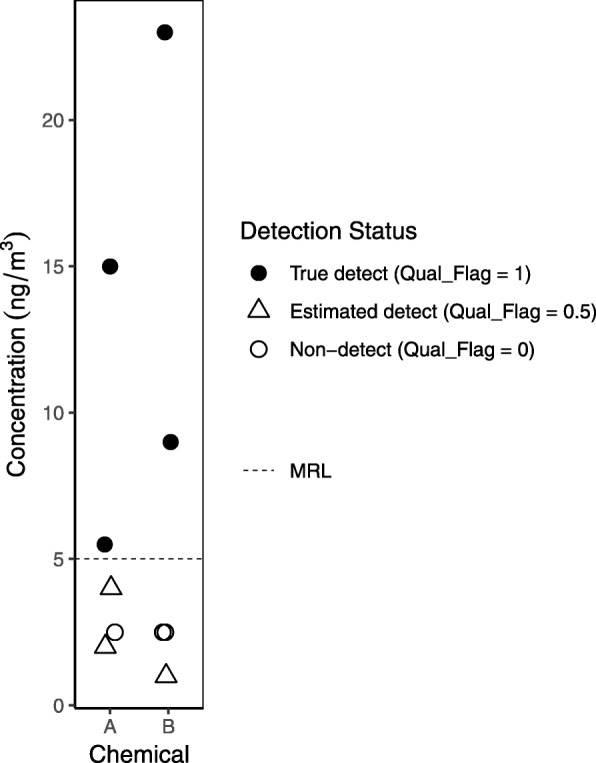
Example of graphical presentation distinguishing true, estimated, and non-detects. MRL = Method Reporting Limit

Typically, we use blanks to qualify values rather than remove measurements from our data. Specifically, we use detected values in field blanks and sometimes other blanks (see Table 6) as a basis to qualify data by raising the method reporting limit (MRL), flagging low values as estimated, until we feel confident in the levels we’re reporting. Values reported by the lab but below the MRL are considered estimated (see Fig. 5 for example of graphical presentation distinguishing estimated detects below the MRL from true detects above the MRL). In the example of the potentially DEHP-contaminated plastic bags used to transport samples, however, we decided not to report DEHP levels for the post-occupancy samples, given the evidence that contamination might have significantly biased the results in that batch. Unexpected findings, such as a chemical or chemicals detected at much higher levels in a lab blank (matrix, solvent method, or other) than in the field blanks, warrant further investigation. In this case, we might suspect that the lab blank was contaminated by another sample; examining the sample run order (which must be requested from the lab, see example correspondence in Additional file 2) could shed light on whether a very high sample was run directly before the lab blank.

**Table 6:** *Consider Raising Method Reporting Limits (MRLs)*

Approach (see Additional file 4 for example of this approach with real data):	
1A. For chemicals not detected in blanks, the MRL is equal to the laboratory reporting limit.	
1B. For each chemical detected in blanks, *if there are detects in blanks in all batches*, establish the MRL as follows (otherwise proceed to 1C):	
□ Compare the lab’s reporting limit to the 90th percentile of field blanks (computed with non-detects set to ½ lab’s reporting limit). The higher value is the new MRL.	
○ However, if we observe many detects in other types of blanks (e.g., matrix, solvent), we consider determining the MRL by comparing the lab’s reporting limit to the 90th percentile of ALL blanks (computed with non-detects set to ½ lab’s reporting limit). The higher value is the new MRL.	
○ It can be helpful here to plot sample data with different possible MRLs to gain understanding of precisely what is being achieved by raising the MRL (i.e., are we successfully flagging data that we are not confident in and at the same time leaving data in which we have confidence unqualified?). See Additional file 4: Figure S9, for an example of this type of plot.	
*Note*: we use the 90th percentile of the blanks rather than using the maximum value or the mean because the 90th percentile is less sensitive to extreme values and can be estimated for data that are not normally distributed. However if the overall study is small (e.g., in our practice, when we have < 5 blanks), we set the MRL equal to the maximum blank mass.	
1C. For each chemical detected in blanks, *if detects in blanks are clustered in one or a few batches*:	
□ If just one extremely problematic batch, consider dropping the sample data from that batch.	
□ If multiple field blanks were run in each batch, can consider determining MRL as above but on a batch-specific basis.	
○ In this case, the way to proceed will very much be a judgment call. Spend time with the data considering various approaches.	
□ Data from reference material and duplicate samples can be helpful in deciding which data points should be qualified because they are “in the noise.”	
2. After determining the MRL, we *flag each sample result* as follows:	
□ 0 flag = measurement reported by the lab as “non-detect”	
□ 0.5 flag = measurement falls below the MRL. These are considered “estimated detects”	
□ 1 flag = measurement falls above the MRL. These are considered “true detects”	
Note that our data qualifier flags may differ from those used by others. For example, NHANES flags non-detects with a “1” and detects with a “0.”	
3. *Normalize MRL.*	
□ If the MRL is determined on a mass basis but sample results are normalized by some factor, such as sample volume, we compute a sample-specific concentration-based MRL by dividing the mass-based MRL by the sample volume.	
Reporting:	
□ We do not count estimated values (0.5 flags) as detects when reporting % > MRL. We do not use estimated detects to calculate summary statistics such as percentiles (see Table shell S2 in Additional file 2).	
□ In summary statistics, we identify any chemicals with greater than 50% estimated detects and add a footnote: “Imprecise quantification for more than 50% of detected values”.	
□ Graphical presentations should distinguish estimated from true detects (e.g., by plotting as different shapes, see Fig. 5).	
□ For reporting in tables, we use median sample volume across samples to convert mass-based MRL to a single concentration-based MRL for each chemical, if applicable.	
□ There are different approaches for incorporating estimated or 0.5 flagged values in statistical analyses, including performing analyses weighted by estimates of the measurement precision below the MRL, or using censored regression methods [23]. However any approach that incorporates estimated values is preferable to procedures that substitute with the DL, ½ DL, zero, or remove these values, a practice which can introduce bias [25].	

After we establish the MRL for chemicals that are detected in blanks, we are confident that levels in samples above that value are true detects and that they are correctly ranked, but there may still be concern about consistent bias in the actual numeric values being reported, both from contamination in the field or lab or from bias in the analytical method. Consistent bias in levels would not be a major concern for ranking individual exposure or comparing groups within a study but is misleading when comparing to levels reported in other studies. For each chemical, we check for evidence of consistent bias across many blanks and correct concentrations reported in summary tables in our papers to reduce this bias (see Table 7).

**Table 7:** Blank correction

Approach (see Additional file 4 for example of this approach with real data):	
1*. Which blanks to use?*	
□ If detects are spread across all types of blanks (e.g., field, solvent method, matrix), we use all blanks for blank correction. Otherwise we use field blanks. We try to keep our blank correction approach consistent with our MRL approach.	
2. *Which chemicals get corrected?*	
□ If > 5 blanks:	
○ For each chemical, we use a one-sided one sample sign test (special case of binomial test with *p* = 0.5) to determine whether the median of blanks is statistically significantly different from zero. True and estimated detects are treated as positive values and non-detects as negative values.	
▪ We blank-correct chemicals with a sign test *p*-value < 0.05.	
▪ However, if the number of blanks is relatively small (10 or fewer) we consider blank correction even when the sign test does not produce a significant result. The sign test does not take into account the magnitude of the levels detected in the blanks nor does it distinguish different types of blanks (i.e., field and lab).	
• For example, if we have 3 field blanks and 4 lab blanks, and we see consistent levels detected across all field blanks and all but one lab blank, we would consider blank correcting even though the sign test would produce *p* > 0.05.	
□ If ≤ 5 blanks (i.e., for a small dataset):	
○ With five or fewer blanks, the sign test will never be significant. In this case, we blank-correct chemicals with 100% detects in blanks.	
3. *Blank correction*:	
□ Calculate the median value of the blanks, with non-detects set to ½ lab’s reporting limit and using all values (i.e., estimated and true detects).	
○ It is useful to pause here and assess the value being used for blank correction. Is it based on an estimated value below the MRL? What will be the percent change in the median, comparing the original to the blank-corrected data?	
□ Subtract median blank value from all sample results.	
□ Subtract median blank value from the MRL (determined as in Table 6).	
Reporting:	
□ We are explicit about whatever procedure we use to decide whether or not to perform blank correction (sign test or other) and about the statistic (e.g., median, mean) and amount used for correction.	
□ Any presentation of measurements (e.g., summary statistics) should use blank-corrected values because they may be compared with measurements in other studies.	
□ For statistical analyses such as regression and correlation performed within the dataset, non-blank-corrected data can be used.	

#### How precise are these measurements?

##### Duplicates

Duplicate samples indicate whether variation in our data is explained by imprecision. If duplicate samples have high reproducibility, meaning that the relative percent difference between measurements in duplicate samples is less than 30%, it adds to confidence in the field sample results. In fact, excellent precision in duplicate samples can influence a decision about how to treat data for a chemical that has sporadic blank contamination or variable spiked sample or CRM recoveries because it can indicate that the results are reproducible. On the other hand, consistently poor precision for dust wipe samples, for example, has informed our decision to rely more heavily on measured air concentrations as an indicator of home exposure [30]. Table 8 outlines our approach for analyzing duplicate data.

**Table 8:** Duplicates

Approach (see Additional file 4 for example of this approach with real data):	
1. *Compute* precision.	
□ Compute & summarize average relative percent difference (RPD) for duplicate pairs or, if ≥3 side-by-side samples, compute relative standard deviation (RSD):	
○ If sample results have been normalized (e.g., mass converted to concentration), compute precision with normalized values.	
○ Compute *only for pairs where both samples are detects.*	
○ Also consider precision restricted to pairs where both samples are flagged as “true detects” above the MRL.	
2. *Visualize* duplicate pairs.	
□ This is a good point to pause and check your data and to note/investigate anything that looks unusual (e.g., huge difference in results for two members of a duplicate pair, how tight are detect/non-detect pairs). See Additional file 4: Figure S10A-D for an example.	
3. *Average* duplicates, with non-detects set to lab’s reporting limit.	
□ Calculate average volume and concentration for each pair. Note, can skip this step if *only* have mass data, or if results were reported by lab as concentrations, rather than as masses that were then normalized to concentrations.	
□ Back calculate new average mass using average volume and average concentration. Or, simply average the duplicate measurements if *only* have mass data, or if results were reported by lab as concentrations, rather than as masses that were then normalized to concentrations.	
□ Compare new average measurement to MRL to determine data qualifier flag.	
□ Combine duplicate averages back with rest of data.	
Reporting:	
□ In publications, we note the range of average RPDs across all chemicals in our QA/QC discussion. We consider average RPD < 30% to be “good” precision.	
□ If a chemical has sporadic blank contamination or variable spike recoveries, excellent precision can increase our confidence in the field sample results.	

#### Publication: how do we tell others about our data?

While it is imperative that a researcher has a thorough understanding of the quality of her own data, it is equally important that she clearly communicate the results of the QA/QC review. When we considered the articles included in our recent review of epidemiologic studies of environmental chemicals and breast cancer [11], we identified gaps in reporting and/or interpretation of QA/QC data, an issue also noted by LaKind et al. [12]. To encourage more regular and consistent reporting of QA/QC results, in supplementary material we provide examples of the tables and plots (Additional file 2: Tables S3, S4, and Figure S1) we have used to communicate QA/QC findings in our publications. Consistently publishing QA/QC findings allows readers to think for themselves about the quality of the data and can inform risk of bias assessments in a systematic review. QA/QC data also provides a basis for determining whether further analyses of the published data (e.g., comparisons to or pooling with other datasets) are appropriate.

## Conclusion

Several real examples from our data demonstrate that close examination of lab and field quality control data is worth the effort. By providing a detailed example of how we have processed and drawn conclusions about our own environmental exposure data (Additional file 4), we aim to make our guidelines explicit and straight forward so that others may adopt and build on them.

## Supplementary information

**Additional file 1: Table S1.** Summary of our application of the Navigation Guide Criteria for Low Risk of Bias Assessment for the question: "Were exposure assessment methods robust?".

**Additional file 2:** Section I: Example lab correspondence. Section II: **Table S2.** Summary statistics table shell. **Table S3.** Quality assurance and quality control (QA/QC) summary table shell. **Table S4.** Findings and actions from review of quality assurance and quality control (QA/QC) data table shell. Section III: **Figure S1.** Distribution of surrogate recoveries by study visit.

**Additional file 3:** Example of report formatting request to send to the lab.

**Additional file 4:** Example QA/QC report.

## Data Availability

Not applicable.

## Abbreviations

CAS: Chemical Abstracts Service
CDC: Centers for Disease Control and Prevention
CRM: Certified reference material
CRQL: Contract required quantitation limit
DEHP: Bis(2-ethylhexyl) phthalate
DL: Detection limit
EPA: United States Environmental Protection Agency
HBCD: 1,2,5,6,9,10-Hexabromocyclododecane
LCS: Laboratory control sample
LOD: Limit of detection
LOQ: Limit of quantitation
LQL: Laboratory quantitation level
MDL: Method detection limit
MRL: Method reporting limit
NIST: National Institute of Standards and Technology
PQL: Practical quantitation limit
PUF: Polyurethane foam
QA/QC: Quality assurance/quality control
QC: Quality control
RL: Reporting limit
RPD: Relative percent difference
RSD: Relative standard deviation
SRM: Standard reference material
