# Supplementary material for: Wrangling environmental exposure data: guidance for getting the best information from your laboratory measurements
Source: Environ Health. 2019 Nov 21;18:99. doi: 10.1186/s12940-019-0537-8 (PMC6868687; doi:10.1186/s12940-019-0537-8)
Supplement: Supplementary file 1 — Additional file 1: Table S1. Summary of our application of the Navigation Guide Criteria for Low Risk of Bias Assessment for the question: "Were exposure assessment methods robust?". [file 12940_2019_537_MOESM1_ESM.pdf]

Table S1. Summary of our application of the Navigation Guide Criteria for Low Risk of Bias Assessment for the question: "Were exposure assessment methods robust?"

First Author	Year	Journal	Measure	Investigation and Prevention of Blanks Contamination	Standards Recovery	Measure of repeatability	Limit of Detection or Quantification Reported	Meets Navigation Guide Criteria for a "Low Risk of Bias" Determination	Comment
Gatto	2007	Cancer Causes Control	OCPs in blood		√	√	√	Yes	
Itoh	2009	Cancer Causes Control	OCPs in blood	√		√	√	Yes	No interpretation of ICC -- what is good/acceptable?
Bonefeld-Jorgensen	2011	Environ Health	PFCs, PCBs, OCPs in blood	√	√		√	Yes?	Link to inter-lab comparison doesn't work; blank contamination investigation for PFCs but not PCBs/DDT.
Recio Vega	2011	J Appl Toxicol	PCBs in blood		√	√	√	Yes	
Boada	2012	Environ Health	OCPs in blood	√	√		√	Yes	Sampling and collection equipment tested for presenence of target chemicals, but no other investigation of blanks .
Bonefeld-Jorgensen	2014	Cancer Causes Control	PFAS in blood		√	√	√	Yes	Standards recovery only for PFOA and PFOS.
Trabert	2014	Cancer Causes Control	BPA in urine	√	√	√		Yes	No investigation of blank contamination, though authors note that measuring conjugated BPA metabolite (BPA-G) reduces concern about contamination.
Ghisari	2014	Environmental Health	PFCs, PCBs, OCPs in blood	√	√		√	Yes?	Follow-up to Bonefeld-Jorgensen, 2011, so just references that paper.
Brauner	2014	Int J Environ Health Res	PCBs in buttock tissue		√	√	√	Yes	
Cohn	2015	J Clin Endocrinol Metab	OCPs in blood	√	√	√	√	Yes	Mentions running method blank, but not the results.
Arrebola	2015	Sci Total Environ	PCBs, OCPS in blood	√	√	√	√	Yes	
Terrell	2016	Occup Environ Med	PBB flame retardants in blood		√	√	√	Yes	
Hurley	2011	Breast Cancer Res Treat	PBDE flame retardants in breast adipose					No	"A full description of laboratory methods and quality control efforts appears elsewhere" but the publication referenced is not easily accessible (e.g., does not show up in PubMed or google search).
Parada	2016	Eur J Cancer	OCPs in blood	√		√		No	CVs without interpretation and range from 35-40% for the lower pool.
Cohn	2007	Environ Health Perspect	OCPs in blood			√		No	CVs without interpretation and one is CV > 20%.
Charlier	2007	Bull Environ Contam Toxicol	OCPs in blood		√		√	No	Inter-lab comparison mentioned, but results not shared.
Iwasaki	2008	Sci Total Environ	OCPs in blood (plasma)			√	√	No	
Yang	2009	Arch Toxicol	BPA in blood				√	No	
Xu	2010	Environ Health Perspect	OCPs in blood		√			No	No recoveries reported but specifies isotope-dilution method. Limits of detection were sample-specific which makes harder to report, but could give a range with median.
Lopez-Carrillo	2010	Environ Health Perspect	Phthalates in blood		√			No	No recoveries reported but specifies isotope-dilution method.
Ociepa-Zawal	2010	Journal of Environmental Science and Health	OCPs in breast (case) and abdominal (control) adipose tissue					No	
Warner	2011	Environ Health Perspect	Dioxins in blood					No	
Cohn	2012	Breast Cancer Research and Treatment	PCBs in blood			√	√	No	
Martinez-Nava	2013	Biomarkers	Phthalates in blood		√		√	No	No recoveries reported but specifies isotope-dilution method.
Tang	2014	Environ International	DDT in soil samples, estimated levels in serum					No	
Holmes	2014	Int J Circumpolar Health	Phthalates, PBDE flame retardants, PCBs, OCPs in blood and urine	√	√			No	No recoveries reported but specifies isotope-dilution method. Discussion of limiting contamination during collection.
Ellsworth	2015	Environmental Research	PCBs in breast tissue	√				No	Mentions duplicates, blanks and spikes but not the results (except for noting blank subtraction, but not the amount).
Yang	2015	Environmental Toxicology and Pharmacology	OCPs in blood and adipose				√	No	
Pastor-Barriuso	2016	Environ Health Perspect	PCBs, OCPS in blood				√	No	
Arrebola	2016	Sci Total Environ	PCBs, OCPS in blood and breast adipose		√		√	No	

Grey shading indicates uncertainty about the adequacy of reporting of that particular measure (see Comment).

OCPs = organochlorine pesticides; PFCs = perfluorinated chemicals; PCBs = polychlorinated biphenyls; PFAS = perfluoroalkylated substances; BPA = Bisphenol A; PBB = polybrominated biphenyls; PBDE = polybrominated diphenyl ethers; DDT = Dichlorodiphenyltrichloroethane

Navigation Guide Criteria for Low Risk of Bias Assessment for the question: "Were exposure assessment methods robust?"

The reviewers judge that there is low risk of exposure misclassification and any one of the following:

• There is high confidence in the accuracy of the exposure assessment methods; or
• Less-established or less direct exposure measurements are validated against well-established or direct methods

AND if applicable, appropriate QA/QC for methods are described and are satisfactory, with at least three of the following items reported, or at least two of the following items reported plus evidence of satisfactory performance in a high quality inter-laboratory comparison: Limit of detection or quantification; standards recovery; measure of repeatability; investigation and prevention of blanks contamination.

From: Johnson PI, Sutton P, Atchley DS, Koustas E, Lam J, Sen S, et al. The Navigation Guide - evidence-based medicine meets environmental health: systematic [illegible]
