# Supplementary material for: Wrangling environmental exposure data: guidance for getting the best information from your laboratory measurements
Source: Environ Health. 2019 Nov 21;18:99. doi: 10.1186/s12940-019-0537-8 (PMC6868687; doi:10.1186/s12940-019-0537-8)

Supplemental Material for:

Wrangling Environmental Exposure Data: Guidance for Getting the Best Information from your Measurements

Julia O. Udesky, Robin E. Dodson, Laura J. Perovich, Ruthann A. Rudel

Contents:

Section I: Example lab correspondence

Section II: Table shells

- Table S2 - Summary statistics table shell
- Table S3 - Quality assurance and quality control (QA/QC) summary data table shell
- Table S4 - Findings and actions from review of quality assurance and quality control (QA/QC) data table shell

Section III: Example figure for communicating QAQC results

- Figure S1 - Distribution of surrogate recoveries by study visit.

I. Example lab correspondence

1. Establishing a relationship with a lab

I'm interested in finding a time to talk about your capabilities for biomonitoring for environmental chemicals.

For our project, we are seeking a laboratory partner to run phthalate metabolites, triclosan, and parabens in urine. We anticipate 100-500 samples over 6 months. I'd also be interested to hear what your services are for biomonitoring for pesticide residues or metabolites - especially in urine.

2. Setting up a contract

Establishing Analytes

We expect to have 50 cotton wipes for our study. The wipes are wetted with isopropyl alcohol prior to collection and samples will be collected from hard surfaces. Attached is the list of analytes we're interested in targeting.

**Compound**	**CAS**
4-Octylphenol	1806-26-4
Octylphenol diethoxylate	9036-19-5
4-Nonylphenol	084852-15-3
…	

Reporting Questions

1. Do you typically run a lab control or matrix spike for each chemical in each batch? If not, please explain. If yes, we would like to request in advance that lab control or matrix spike amounts and recovery amounts be reported along with our sample results.

2. Do you typically run matrix blanks and solvent method blanks? If not, please explain. If yes, we would like to request in advance that matrix blank and solvent blank data be provided along with our sample results.

3. Do you typically blank-correct results prior to reporting?

    - If yes, we would like to request that we also receive files of raw data (before blank correction). This allows us to consider our field blanks in the blank correction process.

    - If it is not possible to provide us with the uncorrected data, what is your procedure for blank correction? Please include as much detail as possible including but not limited to the following:

        - Is correction performed on a lab batch-specific basis?
        - What types of blanks (solvent method, matrix, other) are used for correction?
        - What value is used for correction (e.g. median blank value, other)?
        - Is blank correction performed for estimated values (mass > 0 but less than the lab reporting limit)?

4. What surrogate compounds will be used for determining extraction efficiency? If possible please provide a table indicating which surrogate is being used to represent each targeted chemical.

5. How is the reporting limit determined for each chemical?

6. Do you typically report data qualifier flags?

2/3. Setting up contract/Submitting the first batch of samples to the lab

Reporting Requests:

We have outlined below our data reporting requests as well as some file formatting requests that will make it much easier for us to process the data (see the attached Excel file, **example_reporting.xls**, for an example of ideal data formatting).

A. Data Reporting Requests

(1) When reporting measured values, please:

- Include estimated values when available
- Report non-detects as zeros
- Provide detection limits for all samples, in a separate column. If you use multiple detection thresholds per sample (ex: LODetection and LOQuantitation), include both values in separate columns
- Report values with appropriate significant figures, reflecting the sensitivity of the analytical method
- Use 'NA' when samples could not be analyzed for a particular compound. Do not include any other text values in concentration/amount fields
- Do not perform any blank correction of the data
- If any samples require dilution, please indicate for each chemical what is the final result that should be used
- Include a separate column for flags and a column for any qualifying comments about a particular sample
- Include an indicator for the analytical batch

(2) Please provide flags capturing the following information, as relevant:

**Flag**	**Explanation**
J	estimated value
U	compound was analyzed for, but not detected
B	compound detected in blank as well as sample
D	value is from dilution analysis
I	interference, elevated detection limit
E	value exceeds calibration range
D	value is from dilution analysis
G	imprecise quantification: (e.g. initial calibration non-linearity (%RSD) exceeds 30% or continuing cal. drift exceeds 40%)

(3) QC data

- Please provide:
  - Amount of reference material spiked and amount recovered for each batch, as well as information about any deviations from what is expected
  - Enzyme deconjugation efficiency data for each batch
- If you routinely run any blank samples (e.g. method blanks, batch blanks) please share results with us
- Please provide a unique ID for each QC sample that indicates what kind of sample it is (spike, blank, etc.)
  - To distinguish samples that are split for duplicate analysis, please add a "D" to the end of each duplicate sample ID. For example, "AK-####D"

B. File Formatting Requests

(1) The ideal report (see attached **example_reporting.xls**) includes:

- Sheet 1: General information
- Sheet 2: Data dictionary, including information about flags
- Sheet 3: Measured values in "long format" (see example), beginning with a row of column headers

(2) Please keep chemical names (including capitalization and punctuation), column headers, data qualifier flags (if applicable) sample IDs, reporting of non-detects and estimated detects consistent across data reporting files

(3) Please include the CAS number, if available, as well as the name and configurations (e.g., branched or linear, if relevant) for each chemical analyzed

C. Narrative

- Please provide a narrative that includes information about the materials and methods used in the actual analysis, suitable for publication (e.g. include identity of standards, where standards were purchased). Please also include information on how detection limits should be interpreted.

4. After receiving the first batch of samples from the lab

Would you mind confirming the attached CAS numbers and chemical names with your standards/lab notes? Please let us know if any chemicals were analyzed as a particular isomer (e.g. branched, linear) or mixture of isomers.

Compound	Abbreviation	CAS	Questions
4-Octylphenol	4OP	1806-26-4	branched?
Octylphenol diethoxylate	OP2EO	9036-19-5	
4-Nonylphenol	NP	084852-15-3	
…			

II. Table Shells

Table S2. Summary statistics table shell

	**Pre**							**Post**								
**Analyte**	**No.**[a]	**% > MRL**[b]	**Min.**	**Median**	**GM**[c]	**95th %ile**	**Max.**	**No.**[a]	**% > MRL**[b]	**Min.**	**Median**	**GM**[c]	**95th %ile**	**Max.**	**MRL**[d]	**% Change in median**
(units)																
A [e]		30	--	--												
B [e,f]		20	--	--			[h]									
C [e,g]		100														
D		70	--													
…..																

'--' indicates insufficient number of detects to calculate summary statistic

[a] Number of analyzed samples

[b] MRL = method reporting limit (defined as the maximum of the analytical detection limit and the 90th percentile of the blanks)

[c] GM = geometric mean (specify how non-detects were treated)

[d] Compound-specific MRL, determined using the median volume in the samples

[e] Value subject to blank correction by subtracting the median blank value

[f] Average matrix spike recovery was high (>150%)

[g] Average matrix spike recovery was low (<50%)

[h] Surrogate recovery was high (>150%) for the sample from which we are reporting the maximum value

Table S3. Quality assurance and quality control (QA/QC) summary data table shell

		Blanks								Duplicates			Lab Control Spikes			
**Analyte**	**Blank correct**[a]	**Blank Correct Mass**	**Blank Correct Conc**	**% Change in Median**[b]	**Max Field Blank**[c]	**Max Solvent Method Blank**[d]	**Max Matrix Blank**[e]	**MRL**[f]	**Max MRL**[g]	**N pairs**	**N det pairs**[h]	**Ave RPD**	**N**	**Min % Rec.**	**Max % Rec.**	**Mean % Rec.**
		(units)	(units)		(units)	(units)	(units)	(units)	(units)							
A	yes															80
B	yes															160
C	yes															30
D	no															70
…..																

-- indicates not applicable or not available.

<DL indicates values below the analytical detection or quantitation limit reported by the lab

[a] Concentrations subject to blank correction if the median mass of all of the blanks is significantly different from zero by a sign test

[b] Percent change in the median reported value (if reported) due to blank correction.

[c] Maximum mass detected in field blanks (n =).

[d] Maximum mass detected in solvent method blanks (n =).

[e] Maximum mass detected in matrix blanks (n =).

[f] MRL = method reporting limit defined as (*for example*) the maximum of the analytical detection limit and the 90th percentile of all blanks

[g] A conservative estimate of the MRL based on maximum detected mass in all blanks (field, solvent method and matrix).

[h] Number of unqualified (non-flagged) duplicate pairs with reported values.

[i] Indicates less certainty about reported values due to at least one blank exceeding the median of field samples.

Table S4. Findings and actions from review of quality assurance and quality control (QA/QC) data table shell

QA/QC Measure	Assessed with:	Active Samples
Accuracy	Lab control spikes (Good: 50-150% recovery)	• We ***dropped*** a few compounds (*list analytes*) from further analysis based on poor method performance<br>• Otherwise, across all batches, average recoveries were in range except:<br>○ high: *list analytes*<br>○ low: *list analytes*<br>○ inconsistent*: list analytes*
Extraction efficiency	Surrogate spike in each sample (Good: 50-150% recovery)	• Most surrogate recoveries were within range: 68/84 for surrogate A, 73/84 for surrogate B and 63/84 for surrogate C<br>• Among samples with high/low recoveries, investigation did not show evidence of overestimation or under-reporting
Potential contamination	Field blanks, solvent method blanks, matrix blanks, storage blanks	• *List analytes* were detected in at least one sampler blank at levels greater than the analytical detection limit<br>• *List analytes* were detected in at least one solvent method blank<br>• ***Censored*****:**<br>○ *List analytes* for two batches due to elevated field blanks and lab control spike recoveries<br>***Raised MRL***: *List analytes*
Systematic bias [a]	Consistent detects in the blanks	• ***Blank-corrected***: *List analytes*
Precision	Relative percent difference for side-by-side duplicate samples (Good: < 30%)	• Generally good, except for: *List analytes*

[a] Note that systematic bias and need for blank-correction was assessed *after* censoring certain chemicals from certain batches as noted under ‘potential contamination

<u>III. Example figure for communicating QAQC results</u>

Figure S1. Distribution of surrogate recoveries by study visit.

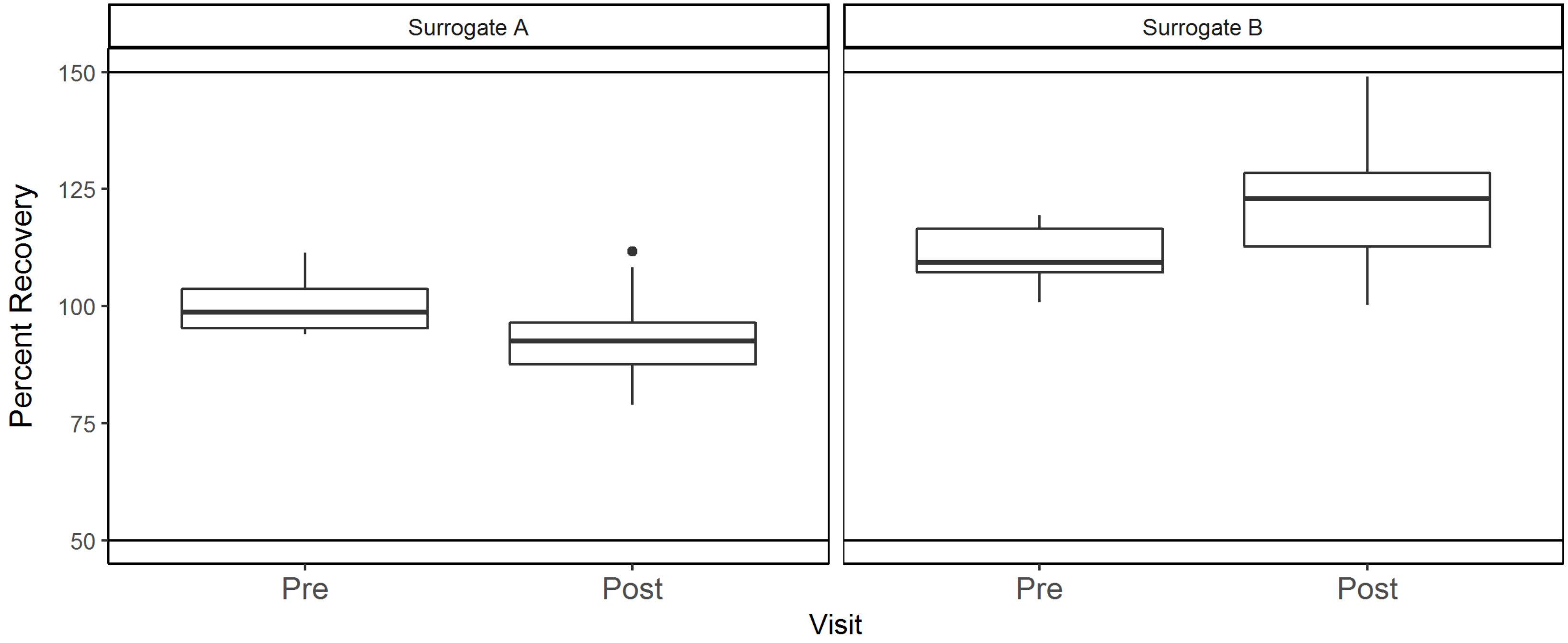

Supplement: Supplementary file 2 — Additional file 2: Section I: Example lab correspondence. Section II: Table S2. Summary statistics table shell. Table S3. Quality assurance and quality control (QA/QC) summary table shell. Table S4. Findings and actions from review of quality assurance and quality control (QA/QC) data table shell. Section III: Figure S1. Distribution of surrogate recoveries by study visit. [file 12940_2019_537_MOESM2_ESM.pdf]
