# Supplementary material for: Wrangling environmental exposure data: guidance for getting the best information from your laboratory measurements
Source: Environ Health. 2019 Nov 21;18:99. doi: 10.1186/s12940-019-0537-8 (PMC6868687; doi:10.1186/s12940-019-0537-8)
Supplement: Supplementary file 4 — Additional file 4: Example QA/QC report. [file 12940_2019_537_MOESM4_ESM.pdf]

xxx Study: QA/QC Report

Contents

Project Description **3**

Section 1. Lab control samples (LCS) **4**

1. *Summarize* LCS percent recoveries for each chemical across analytic batches 4
2. *Visualize* LCS percent recoveries for each chemical across analytic batches to assess consistency 6
3. *Check* if variation in sample results correlates with LCS recoveries by batch 8

LCS Recoveries: Conclusion **9**

Section 2. Surrogate Recoveries **10**

1. *Count* high and low recoveries for each surrogate compound across analytic batches 10
2. *Identify* particular samples where all surrogate recoveries were low, which could suggest a problem with the extraction for that sample. 10
3. *Visualize* surrogate recoveries for QC samples (lab blanks, LCS) across analytic batches 11
4. *Visualize* percent recoveries *across all samples* for each surrogate, overall and by analytic batch 12
5. *Visualize all samples*, colored by associated surrogate recovery 14

Surrogate Recoveries: Conclusion **18**

Section 3.0 Blanks **21**

1. *Summarize* results across all analytes by blank type 21

2._Visualize_ levels in blanks by blank type and analytic batch 26

Section 3.1 Method Reporting Limit (MRL) **28**

1. *List* chemicals never detected in blanks 28
2. *Visualize* levels in blanks compared to levels in samples and consider raising MRL for each chemical detected in blanks 28

Section 3.2 Blank Correction **30**

1. *Which blanks to use?* 30
2. *Which compounds get corrected?* 30

Blanks: Conclusion **31**

Section 4. Duplicates **33**

1. *Compute* precision 33
2. *Visualize* duplicate pairs 35

Duplicates: Conclusion **39**

Project Description

This report summarizes QA/QC findings to support interpretation of results for 39 air samples collected from several indor spaces. Samples were run in 5 analytic batches.

Table 1: Sample type counts by batch. NA means no qc samples of that type were run in the specified batch. Ideally we would have all types of qc samples in each batch.

SampleType	B6	B7	B8	B9	B10
Field Blank	10	6	6	2	2
Field Duplicate	2	16	2	2	NA
Field Sample	14	22	16	16	10
Matrix Blank	NA	2	2	2	NA
Matrix Spike	NA	2	2	2	NA
Solvent Method Blank	4	4	NA	4	2
Storage Blank	NA	NA	NA	4	4

Section 1. Lab control samples (LCS)

Lab control samples were created by spiking known amounts of target analytes into a clean sampler matrix.

1. ***Summarize*** LCS percent recoveries for each chemical across analytic batches

Table 2: LCS Recovery Summary Stats

Abbreviation	N	Min	Mean	Median	Max	Flag
DEP	3	73	87	82	106	
DBP	3	35	86	102	122	
BBP	3	64	88	99	102	
DEHA	3	69	92	102	106	
DEHP	3	68	93	103	109	
DCHP	3	68	91	98	106	
DINP	3	75	165	175	246	High
NP	3	105	139	105	208	
AHTN	3	81	111	115	137	
HHCB	3	78	85	88	90	
MK	3	60	77	81	89	
MX	3	91	114	92	160	
MePa	3	4	5	5	5	Low
BuPa	3	24	31	28	40	Low
BP	3	83	153	88	288	High
BP-3	3	89	144	146	198	
TCS	3	54	82	93	99	
PCB 11	3	63	74	80	80	
PCB 52	3	24	59	75	78	
PCB 153	3	19	65	86	89	
BDE 28	3	78	89	79	111	
BDE 47	3	78	95	95	112	
BDE 99	3	84	111	93	156	
BDE 100	3	67	94	95	120	
23DB1P	3	48	58	62	62	
22BBM13P	3	27	79	53	157	
BEH-TEBP	3	5	58	79	90	
EH-TBB	3	152	274	202	467	High
1C2P	3	77800	1452267	989000	3290000	High
13DC2P	3	30	52	32	93	
TCIPP	3	76	88	81	106	
TDCIPP	3	5	130	176	208	

Abbreviation	N	Min	Mean	Median	Max	Flag
TCEP	3	30	74	84	109	
TBOEP	3	30	131	172	192	
TPHP	3	72	78	74	88	
TBPP	3	68	116	103	177	

Comments:

- **High avg**: BP, DINP, EHTBB, 1C2P
  - 1C2P recovery is extremely high for all LCSs (minimum recovery 77800%). After discussing with lab analyst concluded that method was not accurate for this chemical so we will drop it.
  - For BP, DINP, EHTBB, take a look at LCS recoveries for each individual batch to see if one bad batch is driving the high average.
- **Low avg**: MePa, BuPa
  - For methyl and butyl paraben can see from the summary statistics that recoveries were consistently low across all LCSs. Talk to the lab.

Discussion with lab analyst:

From 8/10/2017 phone call:

- Possible hypothesis for low LCS recoveries was inadequate derivitization by BFTSA. We asked whether we should expect to see the same effect in samples (under-recovery) but analyst noted it would depend what other chemicals were on the samplers (the matrix). She recommended that we look at spike check recoveries and consider correcting LCS recoveries with spike checks. We tried this out and observed that correcting by spike check would improve results for some compounds while introducing more variability for others; in the absence of a clear rule/justification for correcting some compounds but not others, we decided not to correct. We will note in publications that methyl and butyl paraben may be under-reported.

2. *Visualize* LCS percent recoveries for each chemical across analytic batches to assess consistency

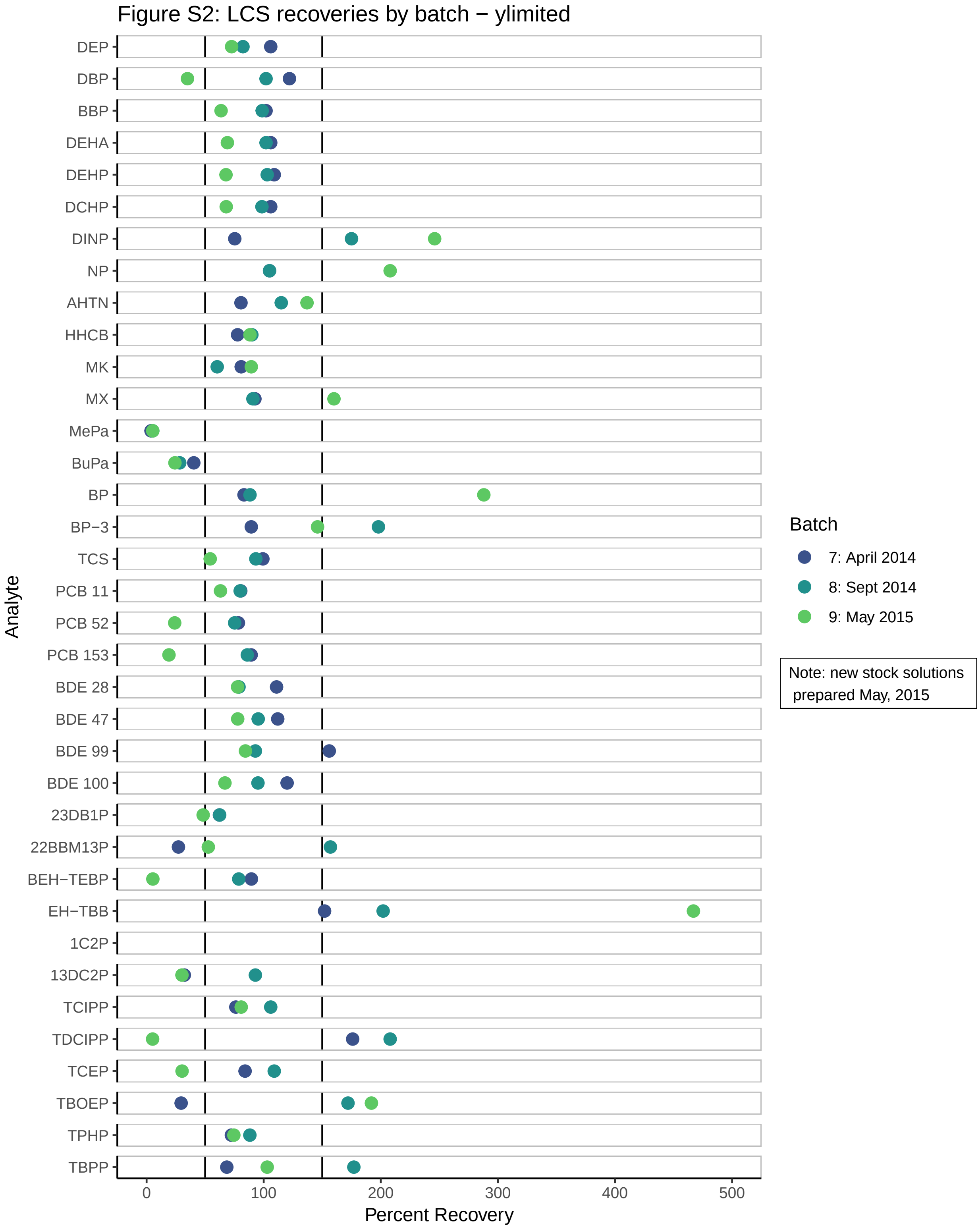

Comments:

- High in all batches: EH-TBB
- High in 2 batches: DINP, TDCIPP, TBOEP
- Inconsistent (some high, some low): 22BBM13P, TDCIPP, TBOEP
- Possible problems with a batch: several chemicals (DINP, NP, BP, EH-TBB, TBOEP) with a high LCS recovery in batch 9.

3. *Check* if variation in sample results correlates with LCS recoveries by batch

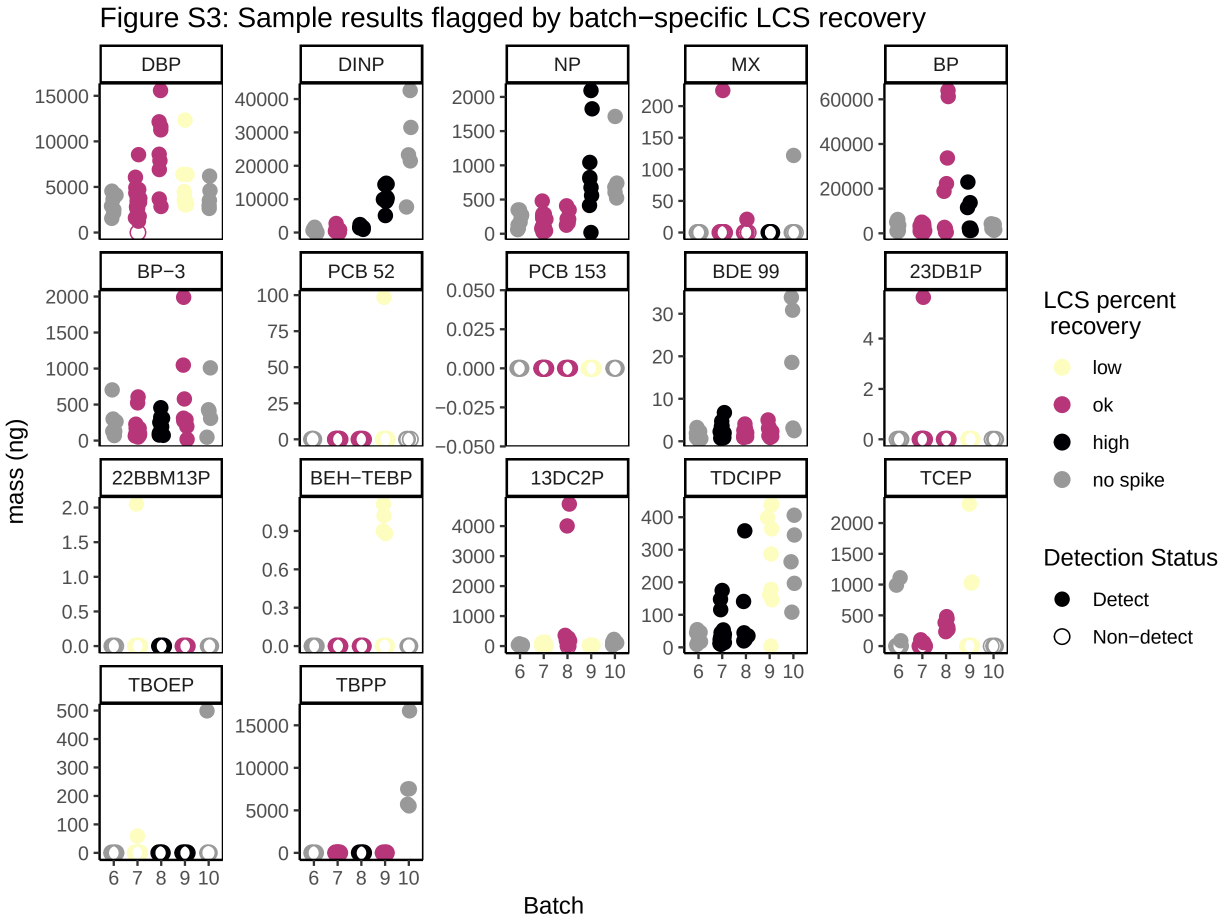

Comments:

- For the most part, variation in levels measured in samples across batches does not appear to track with variation in LCS recoveries. Maybe some concern about 13DC2P because of lower levels in batches with lower recoveries.
- Batch 9 looks problematic – high LCS recovery in a batch with higher sample results – for several chemicals: EHTBB, DINP, NP. There are some high LCS recoveries for batch 8 as well, but the sample data in this batch don't appear to track with the higher recoveries.

LCS Recoveries: Conclusion

- **Drop** the following chemicals:
  - **1C2P**: LCS recoveries are consistently out of range. Per lab analyst: a valid curve was very difficult to obtain. The solvent method blanks and matrix blanks show very high area counts for this peak. In the method demo data, no valid curve was obtained and the recovery was unable to be calculated. In set 1 of the field samples, a curve was created, but the extraction QC showed a peak at the retention time that may or may not be the derivitized 1-chloro-2-propanol.
  - **13DC2P**: because of low LCS recoveries from some batches, and suggestion that sample results might track with variation in recoveries.
- **Consider dropping**
  - **DINP, NP in batch 9**: given over-recovery of LCS spike and higher sample results in this batch. Suggests pssible contamination. Look at blanks too before making decision.
  - **EH-TBB**: LCS recoveries elevated for $>$ 50% of batches (keeping in mind batch 10 didn't have a spike recovery). Also, sample results are highest for batch 9, which had notably higher LCS recovery. Suggests possible contamination. Look at blanks before making decision.
- **Summary statistics** The following chemicals should be flagged in presentation of summary statistics with a note that the average LCS recovery was out of range:
  - BP, BuPa, DINP, EH-TBB, MePa
- **Sensitivity analyses** should be performed for the following chemicals because of a high/low LCS recovery in certain batches for example, by including lab batch as a covariate in regression analyses:
  - 22BBM13P, 23DB1P, BDE 99, BEH-TEBP, BP, BP-3, BuPa, DBP, DINP, EH-TBB, MePa, MX, NP, PCB 153, PCB 52, TBOEP, TBPP, TCEP, TDCIPP

Section 2. Surrogate Recoveries

Four surrogate compounds were used to evaluate extraction efficiency for individual samples.

1. ***Count*** high and low recoveries for each surrogate compound across analytic batches

Table 3: Surrogate Recoveries - Counts

Abbreviation	N.low	N.high	total
BDE 126	0	9	80
d4-DBP	1	11	80
d5-13DC2P	26	5	80
TCS c-13	4	3	80

2. ***Identify*** particular samples where all surrogate recoveries were low, which could suggest a problem with the extraction for that sample.

Table 4: Samples with consistently low surrogate recoveries

**Abbreviation**	**ID**	**SampleType**	**percentrecovery**	**batch**

Comments:

- No samples with low (<50%) recoveries across all three surrogates

3. *Visualize* surrogate recoveries for QC samples (lab blanks, LCS) across analytic batches

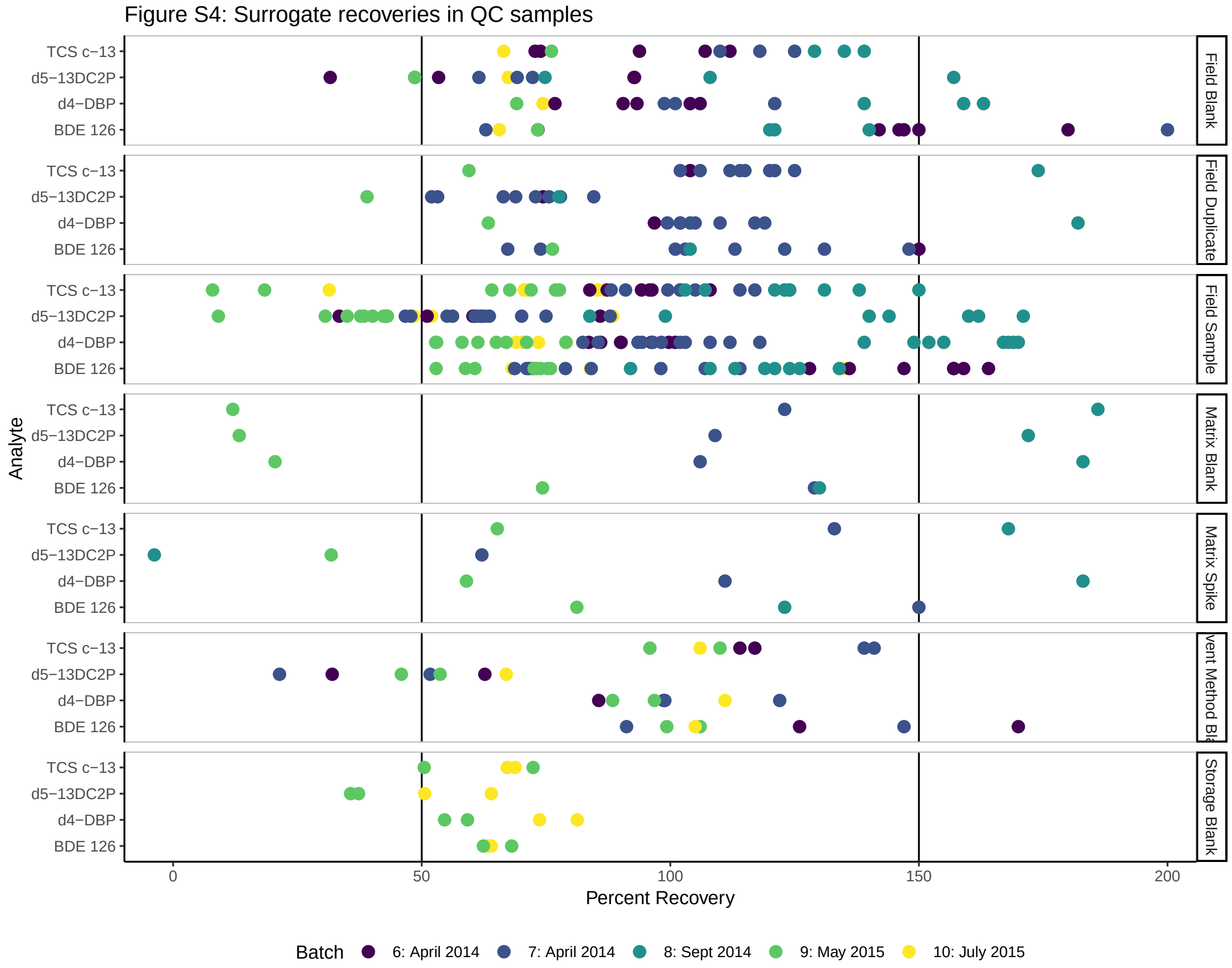

Comments:

- Some batch 8 high surrogate recoveries / batch 9 low, consistent with pattern in samples
- Particularly see this pattern for matrix blank

4. ***Visualize*** **percent recoveries** ***across all samples*** **for each surrogate, overall and by analytic batch**

Figure S5: Surrogate recoveries

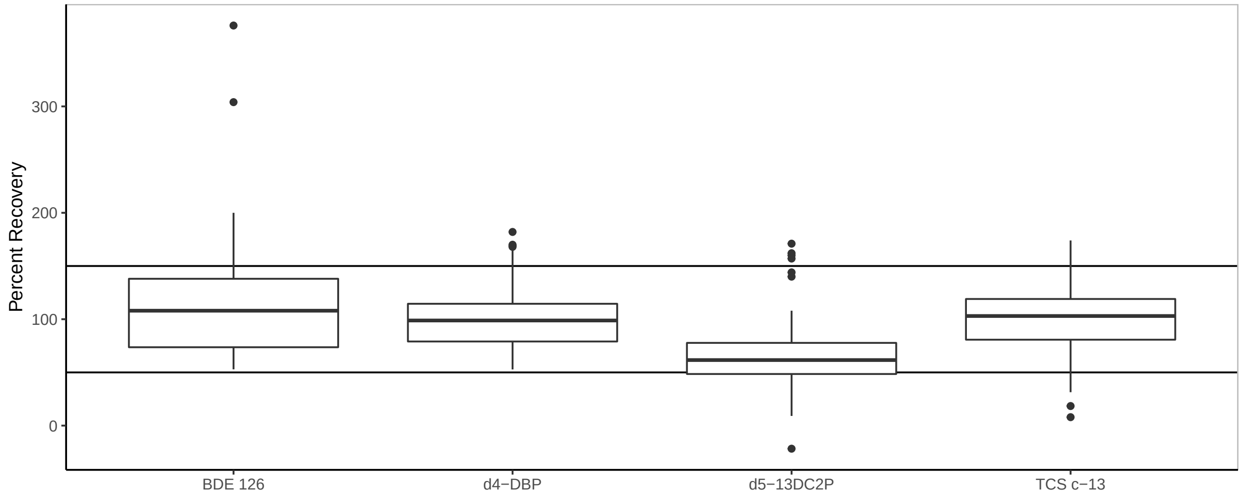

Figure S6: Surrogate recoveries across batches

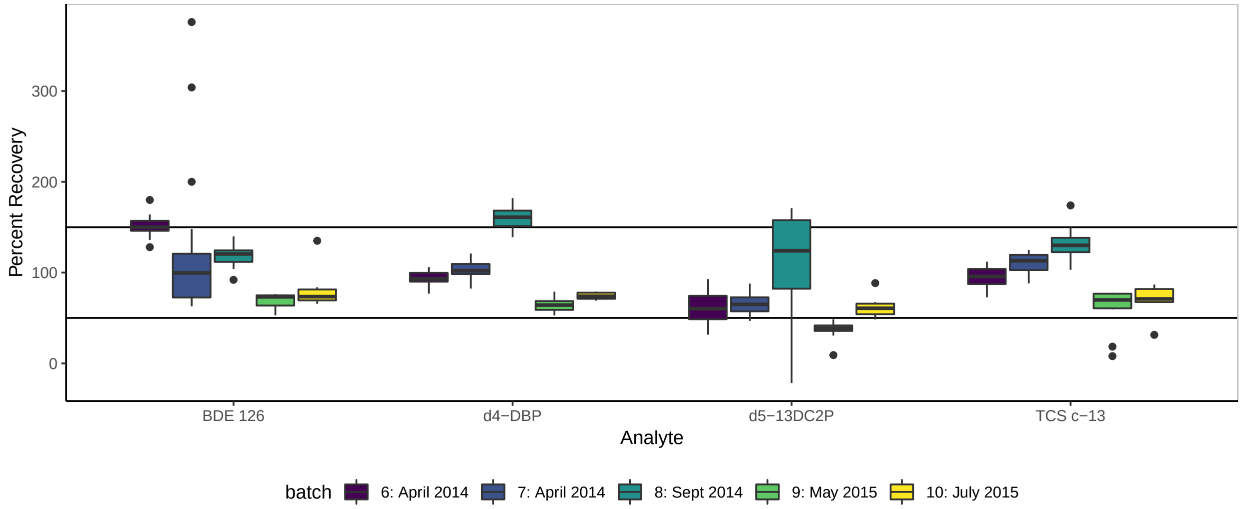

Comments:

- Recoveries are mostly in range except for d5-13DC2P
- There is a noticeable upward drift in the recoveries across analytic batches from April through September 2014 for all surrogates except BDE126. Then we see notably lower recoveries for all four surrogates in batches from May & July 2015.

Discussion with lab analyst:

- We talked to lab analyst about this trend and she gave the following feedback:

  - *The standards used for all batches until May 2015 were the same solutions. New standards were prepared in May 2015. The solutions may have lost some solvent over time between initial preparation and the final batch that was spiked with them. The solutions were stored in a -20C freezer when not in use. In May 2015 some analytes were purchased and all stock solutions were remade as well as the calibration curve and surrogate solutions.'*

- We then asked the lab analyst whether the evaporation off the old standards would affect any other part of the analysis - such as the calibration standards - and whether there would be any way to quantify this potential drift? She directed us to look at the 'spike check' for each batch. The spike check is a vial of solvent that is spiked like the lab control sample, but is neither extracted nor concentrated. It can be treated as a an example of what 100% recovery would look like.
- We looked at spike check recoveries across batches and observed good reproducibility, with no trends to suggest that the drift we saw in surrogate recoveries reflected a problem with the instrument. This adds weight to the potential explanation about solvent evaporation from the stock solutions and gives us confidence that the upward drift for surrogate recoveries does not reflect changes in the instrument calibration over time.

5. ***Visualize all samples***, colored by associated surrogate recovery

Figure S7A: Analytes represented by d4−di−n−butyl−phthalate

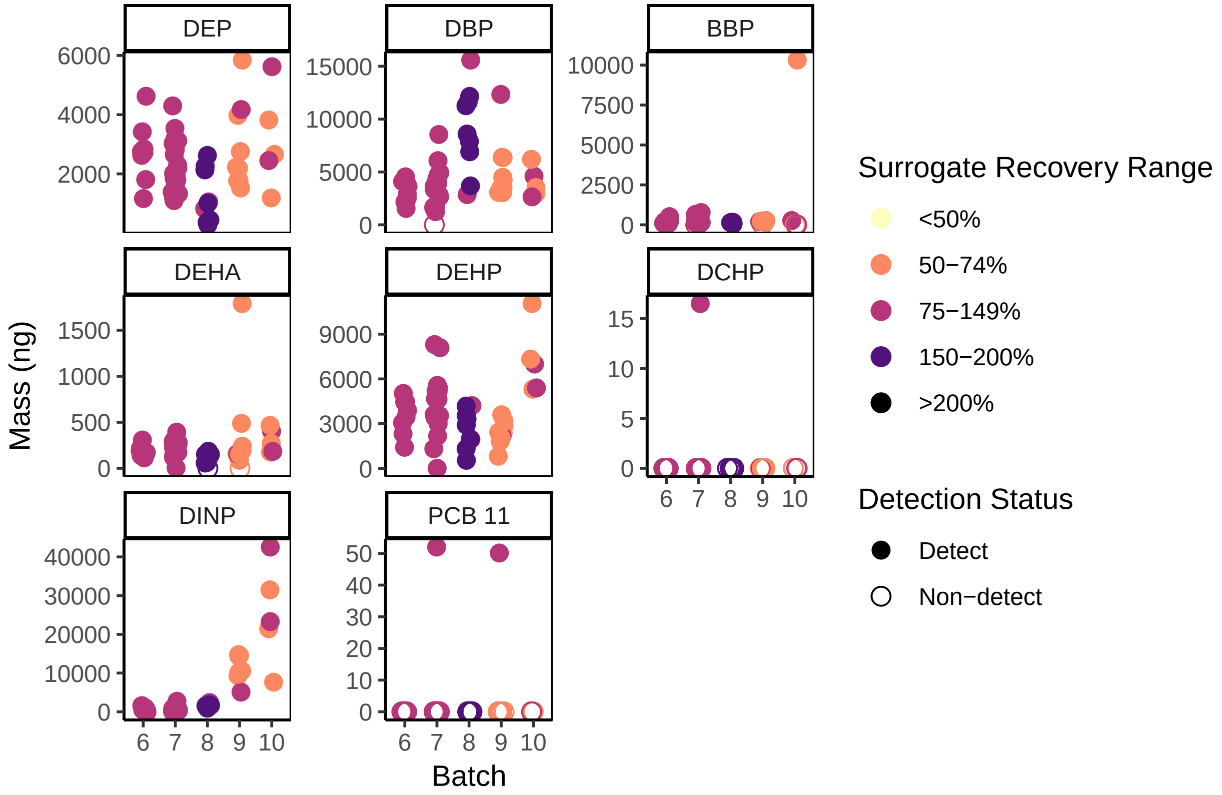

Comments:

- Evidence does not suggest that samples in batch 8, where most had high recoveries of SRS d4-di-n-butyl-phthalate, consistently had the highest levels of the associated analytes.
- No samples had low recoveries for SRS d4-di-n-butyl-phthalate

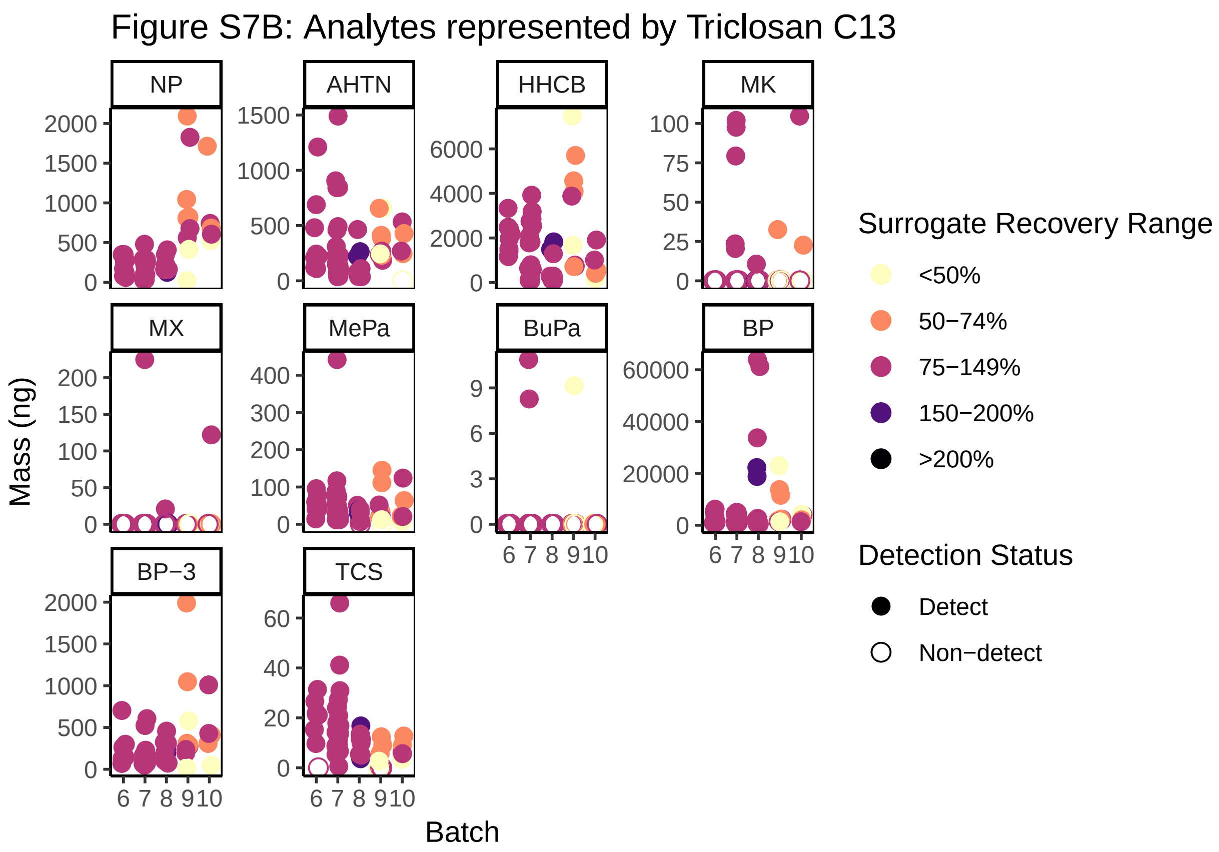

Comments:

- Evidence does not suggest that samples that had high recoveries of SRS triclosan c13 consistently had the highest levels of the associated analytes.
- Evidence does not suggest that samples that had low recoveries of SRS triclosan c13 consistently had the lowest levels of the associated analytes.

Figure S7C: Analytes represented by BDE 126

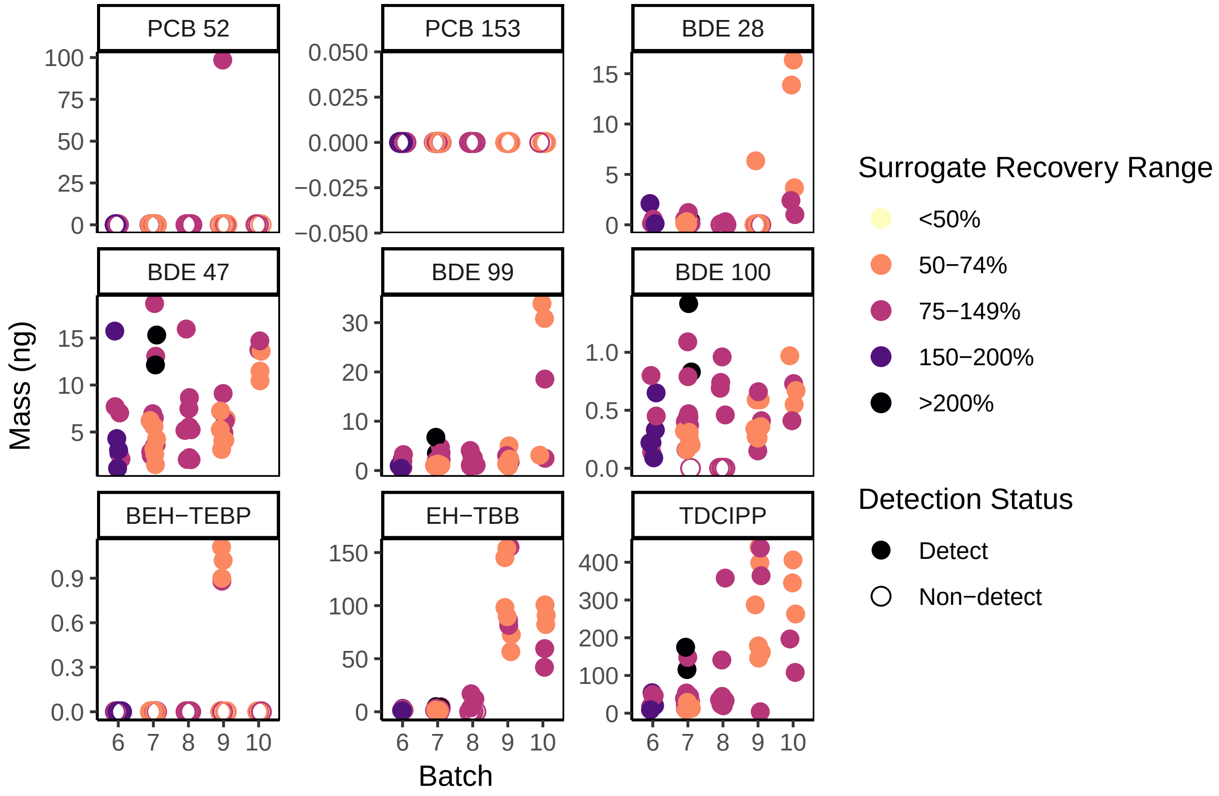

Comments:

- Evidence does not suggest that samples with high recoveries of surrogate recovery standard (SRS) BDE126 consistently have the highest levels of the associated analytes.
- No samples had low recoveries for SRS BDE126.

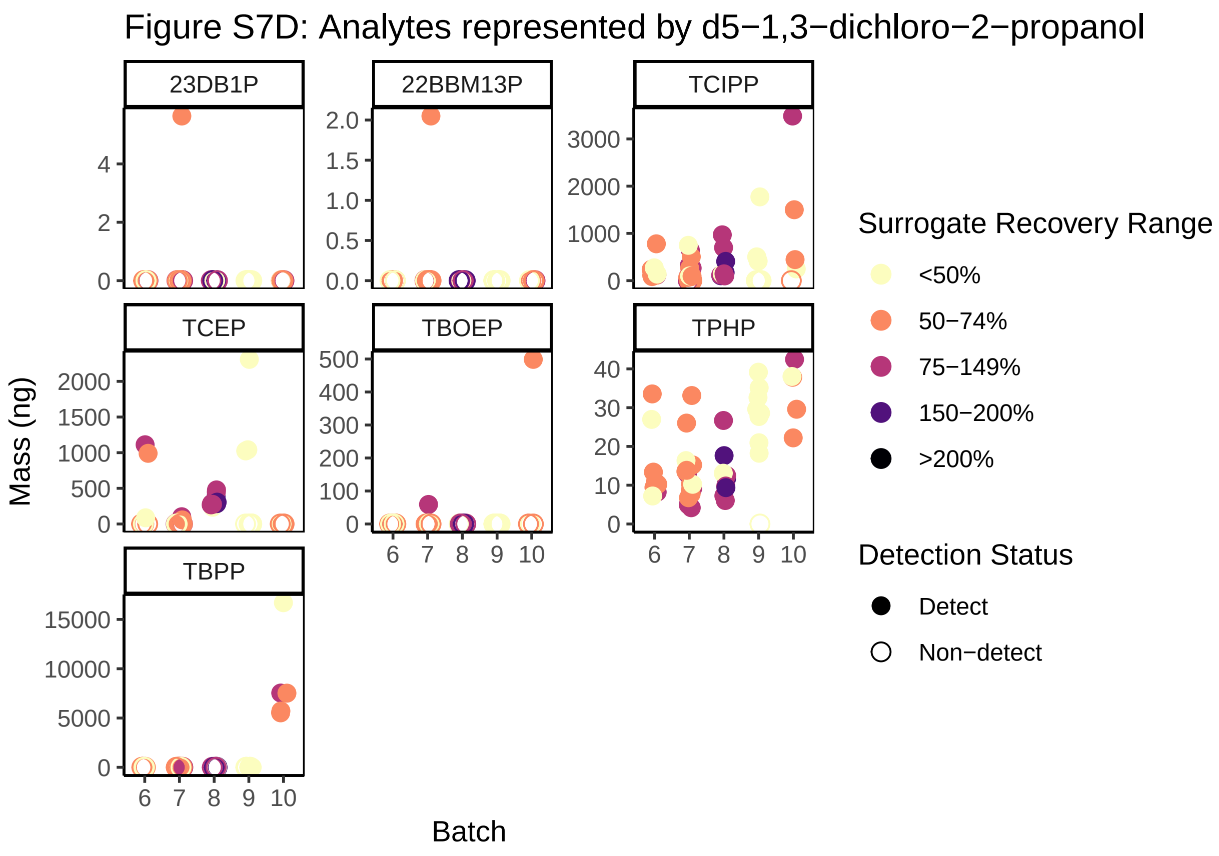

Comments:

- Evidence does not suggest that samples that had high recoveries of SRS d5-1,3-dichloro-2-propanol consistently had the highest levels of the associated analytes.
- Evidence does not suggest that samples that had low recoveries of SRS d5-1,3-dichloro-2-propanol consistently had the lowest levels of the associated analytes.

Surrogate Recoveries: Conclusion

- **Drop** the following samples:
  - Not applicable
- **Perform surrogate correction** for the following samples:
  - Not applicable
- **Summary statistics**
  - The following chemicals should be flagged in presentation of summary statistics with a note that (1) the maximum value is from a sample associated with a high surrogate recovery, and note that in this case the maximum might be overestimated or that (2) the minimum value is from a sample associated with a low surrogate recovery, and note that in this case the minimum might be underestimated:

Table 5: Flag max in summary stats

Abbreviation
BDE 100

Table 6: Flag min in summary stats

Abbreviation
NP
BP-3

- **Sensitivity analyses** should be performed for the following chemicals because of many high/low surrogate recoverys in certain batches for example, by including lab batch as a covariate in regression analyses:

Table 7: Batch sensitivity analysis

Abbreviation	batch	surrFLAG.batch
DBP	8	high
DCHP	8	high
DEHP	8	high
DEP	8	high
DINP	8	high
DEHA	8	high
PCB 11	8	high
BBP	8	high

Abbreviation	batch	surrFLAG.batch
TCIPP	9	low
22BBM13P	9	low
TBPP	9	low
23DB1P	9	low
TCEP	9	low
TBOEP	9	low
TPHP	9	low

- **Sensitivity analyses** should be performed excluding chemicals represented by the surrogate for the following samples because of high / low recoveries of the SRS:

Table 8: Sample sensitivity analysis

ID	surrogate	surrFLAG.samp
665	BDE126 (SRS)	high
6274	BDE126 (SRS)	high
3006	BDE126 (SRS)	high
9681	BDE126 (SRS)	high
6995	BDE126 (SRS)	high
9005	BDE126 (SRS)	high
2898	d4-di-n-butyl-phthalate (SRS)	high
3572	d4-di-n-butyl-phthalate (SRS)	high
3346	d4-di-n-butyl-phthalate (SRS)	high
6440	d4-di-n-butyl-phthalate (SRS)	high
5460	d4-di-n-butyl-phthalate (SRS)	high
2296	d4-di-n-butyl-phthalate (SRS)	high
5715	d4-di-n-butyl-phthalate (SRS)	high
419	d5-1,3-dichloro-2-propanol (SRS)	low
9681	d5-1,3-dichloro-2-propanol (SRS)	low
4865	d5-1,3-dichloro-2-propanol (SRS)	low
7754	d5-1,3-dichloro-2-propanol (SRS)	low
5443	d5-1,3-dichloro-2-propanol (SRS)	high
3346	d5-1,3-dichloro-2-propanol (SRS)	high
6440	d5-1,3-dichloro-2-propanol (SRS)	low
2296	d5-1,3-dichloro-2-propanol (SRS)	high
9160	d5-1,3-dichloro-2-propanol (SRS)	low
2452	d5-1,3-dichloro-2-propanol (SRS)	low
1710	d5-1,3-dichloro-2-propanol (SRS)	low
4137	d5-1,3-dichloro-2-propanol (SRS)	low
9667	d5-1,3-dichloro-2-propanol (SRS)	low

ID	surrogate	surrFLAG.samp
8309	d5-1,3-dichloro-2-propanol (SRS)	low
7321	d5-1,3-dichloro-2-propanol (SRS)	low
4187	d5-1,3-dichloro-2-propanol (SRS)	low
7228	d5-1,3-dichloro-2-propanol (SRS)	low
5376	d5-1,3-dichloro-2-propanol (SRS)	low
3572	Triclosan c-13 (SRS)	high
9667	Triclosan c-13 (SRS)	low
7228	Triclosan c-13 (SRS)	low
468	Triclosan c-13 (SRS)	low

Section 3.0 Blanks

We have lab blanks (matrix and solvent method), field blanks, and storage blanks. Storage blanks are samplers that were sent to us by the lab, stored in our freezer and then returned to the lab for analysis (without ever being opened or going out into the field).

1. *Summarize* results across all analytes by blank type

Table 9: Field Blank Summary Stats. Units: ng. Non-detects are set to zero.

Abbreviation	N	Min	Mean	Median	p95	Max
DEP	13	13.5	156.8	22.42	772	910.8
DBP	13	112.8	457.5	206.9	1260	1574
BBP	13	0	22.94	9.01	84.12	158.7
DEHA	13	0	61.8	66.73	128.5	146.5
DEHP	13	55.87	394.9	84.58	1737	3779
DCHP	13	0	0	0	0	0
DINP	13	0	2212	553.1	10568	16352
NP	13	0	175.3	60.05	842.2	1263
AHTN	13	0	6.669	0	31.47	59.96
HHCB	13	0	6.495	1.7	32.65	45.46
MK	13	0	0	0	0	0
MX	13	0	0	0	0	0
MePa	13	0	0.6892	0	2.212	2.53
BuPa	13	0	0	0	0	0
BP	13	0	71.97	10.68	381.5	429.9
BP-3	13	0	10.63	4.3	35.8	45.35
TCS	13	0	1.201	0	5.638	5.74
PCB 11	13	0	0	0	0	0
PCB 52	13	0	0	0	0	0
PCB 153	13	0	0.4469	0	2.324	5.81
BDE 28	13	0	0.5515	0	2.862	7.11
BDE 47	13	0	0.5892	0.35	1.848	3.45
BDE 99	13	0	2.175	0.2	10.58	25.17
BDE 100	13	0	0.06769	0	0.28	0.34
23DB1P	13	0	0	0	0	0
22BBM13P	13	0	0	0	0	0
BEH-TEBP	13	0	0.06385	0	0.332	0.83
EH-TBB	13	0	13.17	0	70.44	82.93
13DC2P	13	0	0	0	0	0

Abbreviation	N	Min	Mean	Median	p95	Max
TCIPP	13	0	0	0	0	0
TDCIPP	13	0	17.52	8.12	77.83	91.13
TCEP	13	0	86.11	0	384.4	690.1
TBOEP	13	0	0	0	0	0
TPHP	13	0	3.909	1.8	12.57	16.52
TBPP	13	0	2222	0	11553	28882

Table 10: Solvent Method Blank Summary Stats. Units: ng. Non-detects are set to zero.

Abbreviation	N	Min	Mean	Median	p95	Max
DEP	7	1.48	7.539	8.37	13.35	14.81
DBP	7	42.44	161.3	125.7	295.4	307.3
BBP	7	0	3.104	0	11.24	13.17
DEHA	7	0	0.9171	0	3.458	3.83
DEHP	7	13.32	49.24	50.41	95.48	109.6
DCHP	7	0	0	0	0	0
DINP	7	0	65.29	0	278.2	352.8
NP	7	0	0.09714	0	0.476	0.68
AHTN	7	0	0.1214	0	0.595	0.85
HHCB	7	0	0.1686	0	0.702	0.87
MK	7	0	0	0	0	0
MX	7	0	0	0	0	0
MePa	7	0	0.2471	0	0.879	0.9
BuPa	7	0	0	0	0	0
BP	7	0	9.5	3.86	29.58	31.69
BP-3	7	0	2.289	1.08	6.139	6.34
TCS	7	0	0.04714	0	0.231	0.33
PCB 11	7	0	0	0	0	0
PCB 52	7	0	0	0	0	0
PCB 153	7	0	0	0	0	0
BDE 28	7	0	0.005714	0	0.028	0.04
BDE 47	7	0	0	0	0	0
BDE 99	7	0	9.967	0	48.61	69.21
BDE 100	7	0	0	0	0	0
23DB1P	7	0	0	0	0	0
22BBM13P	7	0	0	0	0	0
BEH-TEBP	7	0	0	0	0	0
EH-TBB	7	0	0.01714	0	0.084	0.12

Abbreviation	N	Min	Mean	Median	p95	Max
13DC2P	7	0	0	0	0	0
TCIPP	7	0	0	0	0	0
TDCIPP	7	0	0.09	0	0.365	0.44
TCEP	7	0	0.19	0	0.931	1.33
TBOEP	7	0	0	0	0	0
TPHP	7	0	0	0	0	0
TBPP	7	0	0	0	0	0

Table 11: Matrix Blank Summary Stats. Units: ng. Non-detects are set to zero.

Abbreviation	N	Min	Mean	Median	p95	Max
DEP	3	25.74	386.9	89.3	950	1046
DBP	3	0	153.7	224.8	235.3	236.4
BBP	3	0	66.94	26.98	159.2	173.8
DEHA	3	0	0	0	0	0
DEHP	3	0	201.4	238.8	352.8	365.5
DCHP	3	0	0	0	0	0
DINP	3	270.2	4750	1217	11608	12762
NP	3	26.25	116.6	29.94	267.3	293.7
AHTN	3	0	1.087	0	2.934	3.26
HHCB	3	0	1.41	1.36	2.719	2.87
MK	3	0	0	0	0	0
MX	3	0	0	0	0	0
MePa	3	0	0.7633	0	2.061	2.29
BuPa	3	0	0.2867	0	0.774	0.86
BP	3	4.95	198.3	27.81	508.6	562
BP-3	3	0	15.59	3.14	39.59	43.64
TCS	3	0	2.07	0	5.589	6.21
PCB 11	3	0	0	0	0	0
PCB 52	3	0	0	0	0	0
PCB 153	3	0	0	0	0	0
BDE 28	3	0	0	0	0	0
BDE 47	3	0	0.7033	0.65	1.379	1.46
BDE 99	3	0	0.2033	0	0.549	0.61
BDE 100	3	0	0	0	0	0
23DB1P	3	0	0	0	0	0
22BBM13P	3	0	0	0	0	0
BEH-TEBP	3	0	0.7367	0	1.989	2.21

Abbreviation	N	Min	Mean	Median	p95	Max
EH-TBB	3	0	39.88	0	107.7	119.7
13DC2P	3	0	0	0	0	0
TCIPP	3	0	0	0	0	0
TDCIPP	3	0	6.15	2.76	14.4	15.69
TCEP	3	0	0	0	0	0
TBOEP	3	0	0	0	0	0
TPHP	3	0	7.67	3.74	17.72	19.27
TBPP	3	0	3.117	0	8.415	9.35

Table 12: Storage Blank Summary Stats. Units: ng. Non-detects are set to zero.

Abbreviation	N	Min	Mean	Median	p95	Max
DEP	4	1293	1560	1455	1955	2040
DBP	4	910.5	1908	2122	2470	2479
BBP	4	0	42.28	0	143.7	169.1
DEHA	4	0	31.27	0	106.3	125.1
DEHP	4	439.9	2803	2087	6164	6599
DCHP	4	0	0	0	0	0
DINP	4	8483	16168	17061	22048	22067
NP	4	485.5	1547	1501	2626	2703
AHTN	4	0	66.09	61.09	139.2	142.2
HHCB	4	51.49	63.21	62.33	75.92	76.68
MK	4	0	3.25	0	11.05	13
MX	4	0	0	0	0	0
MePa	4	0	2.473	2.47	4.949	4.95
BuPa	4	0	0	0	0	0
BP	4	545.2	642.7	631.3	744.6	763.2
BP-3	4	20.14	76	60.64	152.5	162.6
TCS	4	0	3.985	4.875	6.138	6.19
PCB 11	4	0	0	0	0	0
PCB 52	4	0	0	0	0	0
PCB 153	4	0	0	0	0	0
BDE 28	4	0	8.515	3.705	23.76	26.65
BDE 47	4	1.11	1.73	1.545	2.591	2.72
BDE 99	4	0.75	22.25	23.1	41.87	42.02
BDE 100	4	0.15	0.2175	0.22	0.28	0.28
23DB1P	4	0	0.29	0	0.986	1.16
22BBM13P	4	0	0	0	0	0

Abbreviation	N	Min	Mean	Median	p95	Max
BEH-TEBP	4	0	0.2725	0	0.9265	1.09
EH-TBB	4	105.1	125.7	117.8	157	161.9
13DC2P	4	0	6.9	6.065	14.97	15.47
TCIPP	4	0	0	0	0	0
TDCIPP	4	98.99	142.9	124.8	209.9	223.2
TCEP	4	0	0	0	0	0
TBOEP	4	0	0	0	0	0
TPHP	4	0	6.375	0	21.67	25.5
TBPP	4	0	6949	6475	14559	14844

2.__Visualize__ levels in blanks by blank type and analytic batch

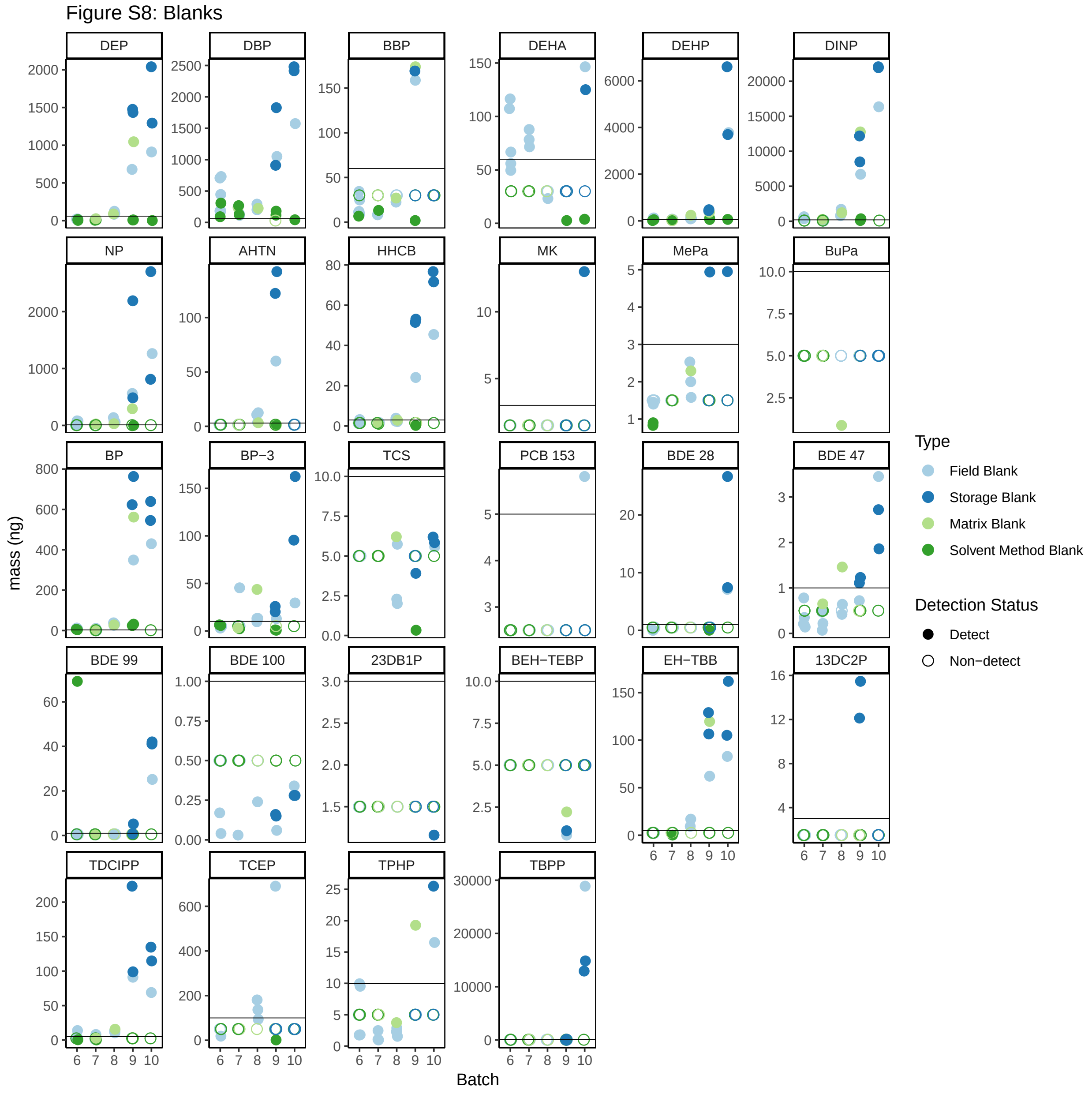

Note that in the above plots, non-detects are set to 1/2 the lab's reporting limit.

Comments:

- Lots of detects in storage blanks. This is hard to investigate further because unfortunately we didn't have storage blanks in batches 6-8.
- Also lots of detects in field and matrix blanks for batches 9 and 10 – DEP, DEHP, DINP, NP, BDE 28, BDE 99, BDE 47, EH-TBB, TDCIPP, TCEP, TPHP, TBPP.

Next step – see how levels in blanks compare to levels in field samples and decide whether it is necessary to raise the Method Reporting Limit (MRL).

Section 3.1 Method Reporting Limit (MRL)

1. *List* chemicals never detected in blanks

DCHP, *MX*, *PCB 11*, *PCB 52*, *22BBM13P*, *TCIPP* and *TBOEP*

2. *Visualize* levels in blanks compared to levels in samples and consider raising MRL for each chemical detected in blanks

Figure S9: Blanks & samples

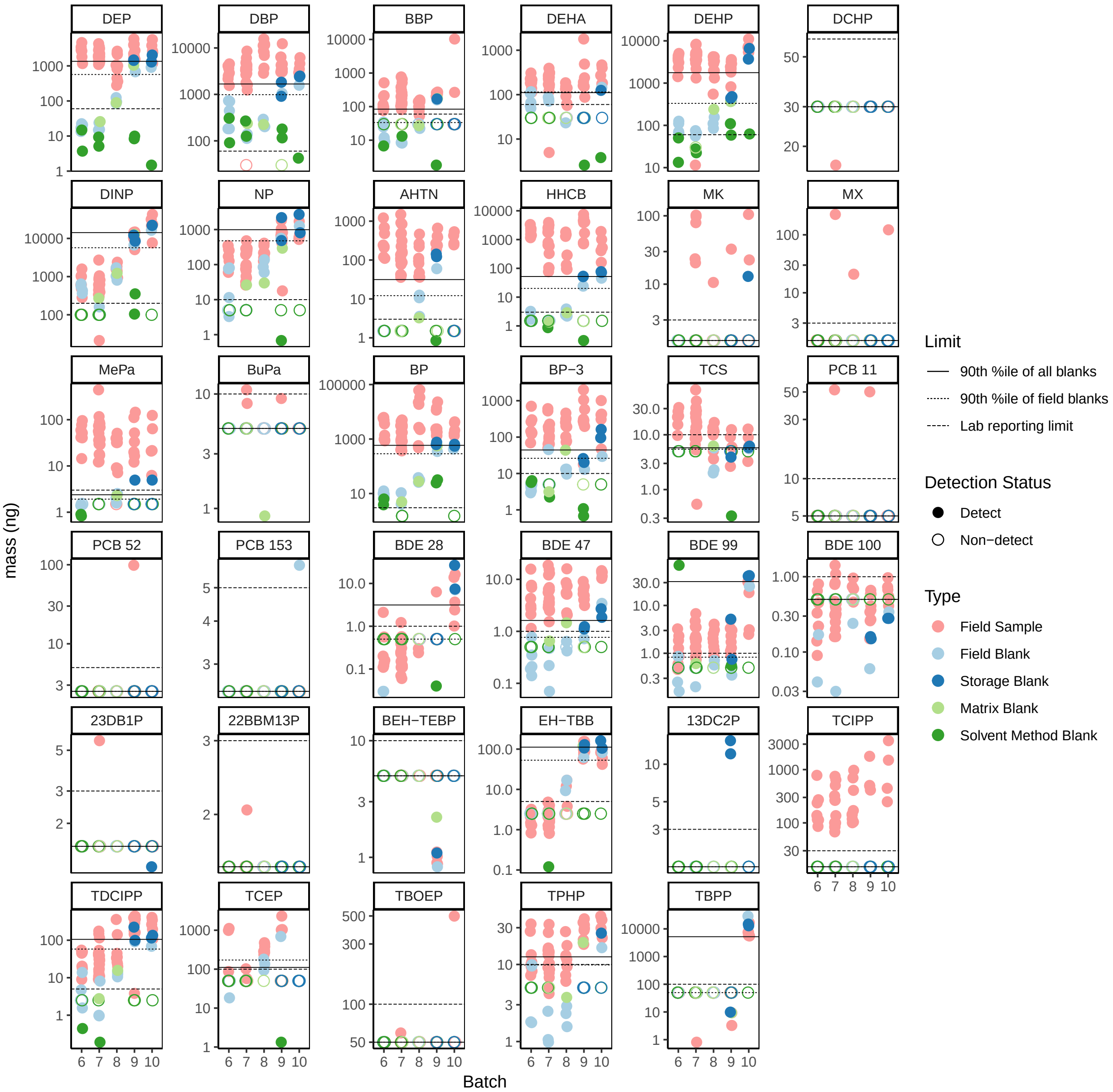

Note: Non-detects are plotted at half lab's reporting limit.

Comments:

- Review of our data indicates that we should use 90th percentile of ALL blanks rather than just field blanks, given that storage blanks are often highest, and we only have storage blanks from 2 batches - seems inappropriate to make a batch-specific MRL (because we would be assuming that storage blanks would have been lower if they had been run in other batches, which we don't have a basis for assuming). Some detects in solvent method and matrix blanks as well. After determining the final MRL by comparing the 90th percentile of ALL blanks to the lab reporting limit and selecting the higher value, we will flag each sample result as follows:
  - **0 flag** = measurements reported by the lab as "non-detect"
  - **0.5 flag** = measurements falling below the MRL. These are considered "estimated detects"
  - **1 flag** = measurements falling above the MRL. These are considered "true detects"
- Based on this plot, we also determined that EH-TBB should be dropped from further analysis. This FR was almost never detected > lab detection limit in batches 6 and 7. In batches 8-10, majority of samples were detects, but field/storage blanks show obvious overlap with sample results.
- We will also exclude all data from batch 9 for DINP and nonylphenol, given problematic lab cotrol sample recoveries and blanks in range of samples in this batch.

Section 3.2 Blank Correction

1. *Which blanks to use?*

- Since detects were spread across all types of blanks (e.g. field, solvent method, matrix, storage), we should use all blanks for blank correction.

2. *Which compounds get corrected?*

- Because we have > 5 blanks, for each chemical, we can use a one sample sign test to determine whether the median of blanks is statistically significantly different from zero. Compounds with sign test p-value < 0.05 get blank-corrected.

Blanks: Conclusion

- We raised the MRL for the following compounds, based on the 90th percentile of ALL blanks:

Table 13: Compound MRLs & Detection Frequencies. Note: % detected includes estimated detects.

Abbreviation	MRL	% detected	% > MRL
DEP	1350	100	78
DBP	1680	98	90
BBP	84	90	80
DEHA	111	96	86
DEHP	1760	100	88
DCHP	60	2	0
DINP	14200	98	10
NP	992	100	2
BuPa	10	6	2
BP	587	100	90
BP-3	44	100	98
TCS	10	88	52
BDE 28	3	70	8
BDE 47	2	100	96
BDE 99	32	100	2
BDE 100	1	88	4
22BBM13P	3	2	0
BEH-TEBP	10	8	0
TDCIPP	105	100	36
TCEP	111	34	28
TBOEP	100	4	2
TPHP	13	98	58
TBPP	5210	16	10

- We will not use estimated detects below the MRL to calculate summary statistics such as percentiles.
- In summary statistics, the following chemicals will be flagged with a footnote: "Imprecise quantification for more than 50% of detected values" given greater than 50% estimated detects.
  - Not applicable.
- We blank-corrected results for six chemicals. Any presentation of actual measurement (e.g. summary statistics) will be based on blank-corrected values. For statistical analyses performed within the dataset, non-blank-corrected data can be used.

Table 14: Compounds to blank correct. Units = ng. Median computed with non-detects set to 1/2 lab's reporting limit.

Abbreviation	median_blank
BP	12.41
BP-3	5
DBP	224.8
DEHP	84.58
DEP	22.42
TDCIPP	2.76

Section 4. Duplicates

We collected 9 side-by-side duplicate pairs and one triplicate.

1. *Compute* precision

Table 15: Precision Summary Stats. Only includes pairs where both samples were detects (true or estimated). Precision is calculated either as Relative Percent Difference (for duplicates) or as Relative Standard Deviation (for triplicates)

Abbreviation	Npairs	minPREC	meanPREC	medianPREC	maxPREC	spikeRSD
TCEP	6	6.85	11.45	11.5	16	54
HHCB	21	2.05	12.85	15.9	28.1	7.77
MK	2	13.5	13.5	13.5	13.5	19.5
AHTN	21	0.222	13.63	8.45	32.1	25.6
BP	21	0.444	15.71	14.7	52.2	76.3
DEP	21	4.33	16.05	11.7	31.7	19.8
DBP	21	3.11	16.72	15.1	37.6	52.9
BP-3	21	0.218	19.18	21	34	37.6
NP	19	8.65	19.53	22.2	36.1	42.7
MePa	21	0.368	27.13	19.2	135	17.7
BDE 100	17	0	36.31	36	85.7	28.3
TPHP	19	9.9	37.04	29.5	83.2	10.9
BBP	19	0.762	41.49	48.3	89.9	24.2
BDE 99	21	0.514	43.33	50.4	92.7	35.2
TCIPP	15	0.132	46.29	17	173	18.3
DEHA	17	0.163	47.25	33	183	22
TDCIPP	21	0.794	49.66	54.6	109	84.1
BDE 47	21	4.15	52.88	71.2	114	18
BDE 28	17	8.7	54.84	58.8	115	21.2
TCS	17	27.1	56.21	46.4	129	29.7
DEHP	21	1.67	60.31	37.2	197	23.9
DINP	17	24.3	62.09	35.1	172	51.8

Table 16: Precision Summary Stats. Only includes pairs where both samples were TRUE detects. Precision is calculated either as Relative Percent Difference (for duplicates) or as Relative Standard Deviation (for triplicates)

Abbreviation	Npairs	minPREC	meanPREC	medianPREC	maxPREC	spikeRSD
TCEP	4	6.85	9.175	9.175	11.5	54
TPHP	4	9.9	10.05	10.05	10.2	10.9
TDCIPP	4	8.99	12.8	12.8	16.6	84.1
HHCB	21	2.05	12.85	15.9	28.1	7.77
MK	2	13.5	13.5	13.5	13.5	19.5
AHTN	21	0.222	13.63	8.45	32.1	25.6
TCIPP	12	0.132	14.61	11.28	36.1	18.3
BP	18	0.444	15.87	11.5	52.2	76.3
DBP	17	3.11	15.96	15.1	37.6	52.9
DEP	16	4.77	18.34	18.05	31.7	19.8
BP-3	21	0.218	19.18	21	34	37.6
BDE 100	2	26.3	26.3	26.3	26.3	28.3
MePa	21	0.368	27.13	19.2	135	17.7
DEHA	15	0.163	29.15	28.2	78.9	22
DEHP	17	1.67	34.02	18.8	115	23.9
BBP	14	0.762	37.4	47.3	89.9	24.2
TCS	8	34.5	43.65	40.65	58.8	29.7
BDE 47	17	4.15	45.45	49.5	114	18

Table 17: Chemicals with true detect / non-detect pairs

Abbreviation	N pairs
BBP	1
DEHA	2
MK	1
MX	1
BuPa	1
TCS	1
PCB 11	1
TCIPP	3
TCEP	1
TPHP	1

2. *Visualize* duplicate pairs

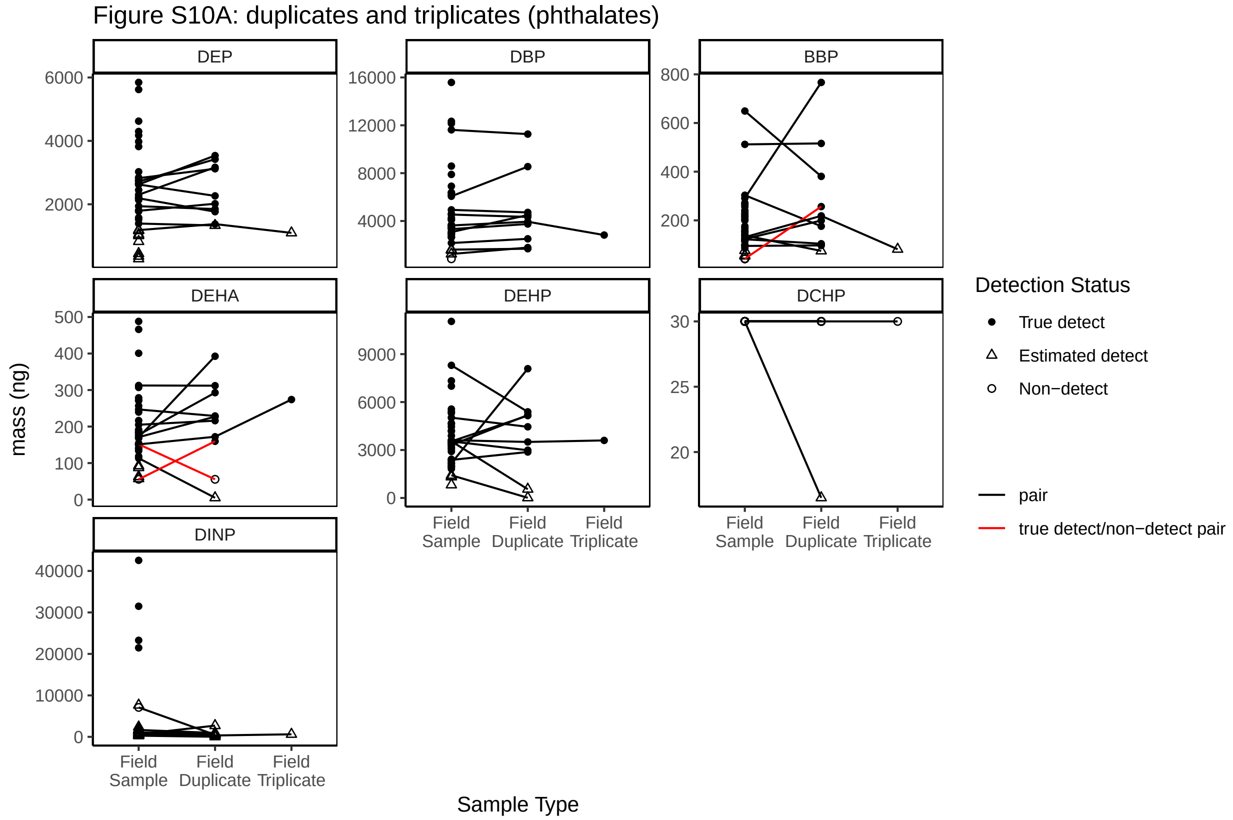

Note, in the plots above, a very high value for DEHA and a very high value for BBP have been censored from the plots in order to view data at lower levels. None of the censored points were part of a duplicate/triplicate set.

Comments:

- These plots reflect what we learned from precision calculation – good precision for DEP & DBP, relatively poor precision for BBP, DEHA, DEHP, DINP.

Figure S10B: duplicates and triplicates (personal care product chemicals)

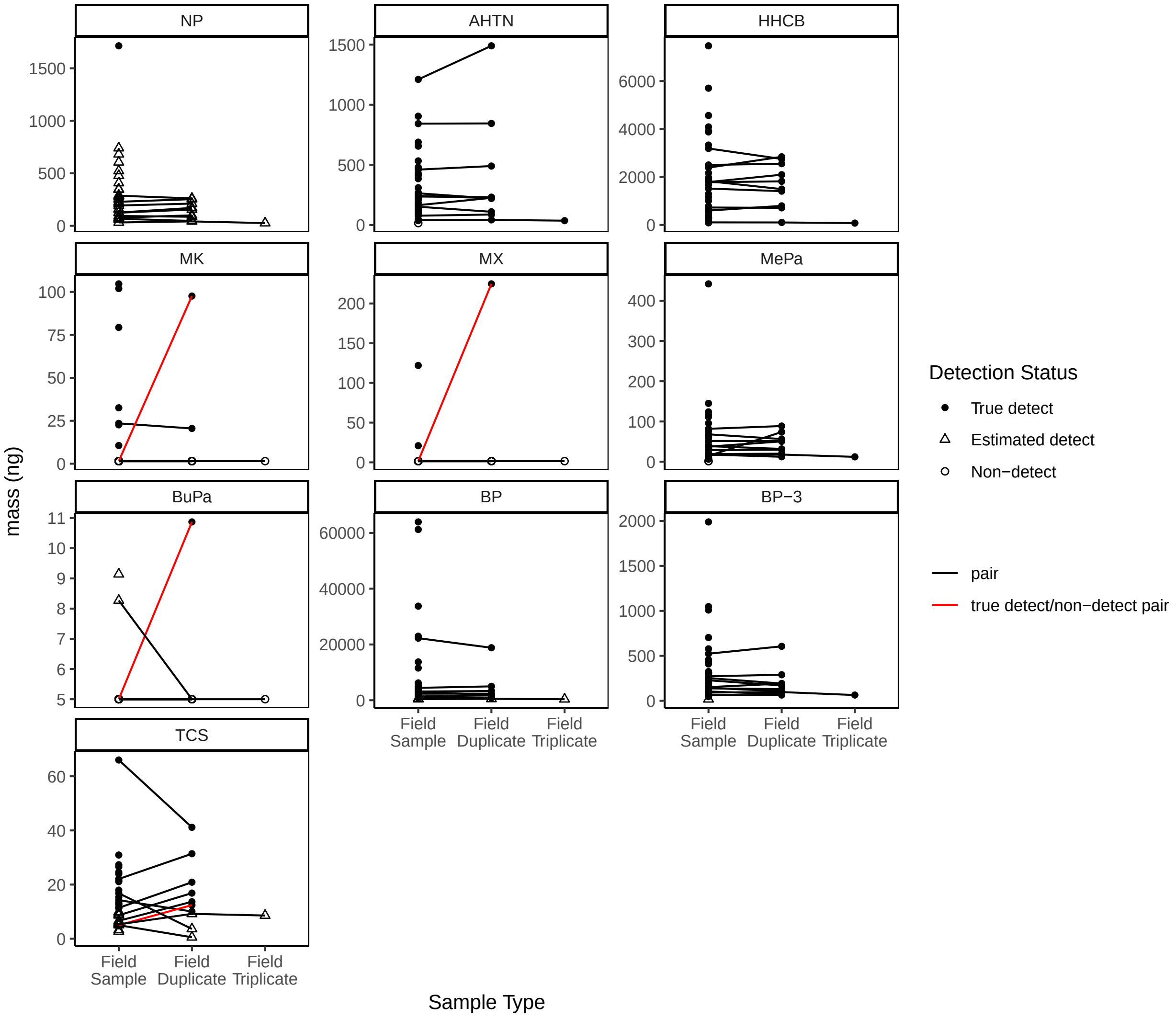

Comments:

- Are the true detect/non-detect pairs for MX, BuPa, MK all the same pair? Yes. Anything unusual about that sample?
  - There are notes in the field log about a leaky window and about some uncertainty about which sample was the field sample and which the field duplicate. These notes don't particularly point to a problem with the sample.
  - Surrogate recoveries for TCS c13 (the representative surrogate for this group) were in the acceptable range for both the sample and dup.
  - The field sample was analyzed in batch 6 and the field duplicate in batch 7. We can't compare LCS recoveries because no LCS was run for batch 6. But blanks (field and lab) look similar between these batches.

No further action indicated at this time.

Figure S10C: duplicates and triplicates (PCBs)

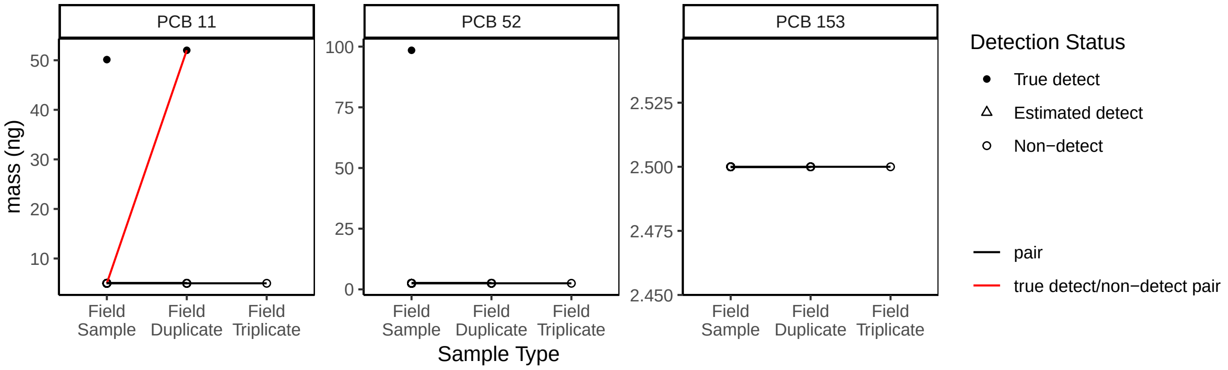

Comments:

- PCBs rarely detected so not much to say about precision.
- One pair imprecise (non-detect and detect pair) for PCB11. Anything unusual about that sample?
  - Not in field log notes, it is the same sample as above (leaky window, uncertainty about field sample vs. duplicate).
  - Surrogate recoveries for d4-di-n-butyl-phthalate (the representative surrogate for PCB 11...) in the acceptable range for both the sample and dup.
  - As noted above, the field sample was analyzed in batch 6 and the field duplicate in batch 7. We can't compare LCS recoveries because no LCS was run for batch 6. But blanks (field and lab) look similar between these batches.

No further action indicated at this time.

Figure S10D: duplicates and triplicates (flame retardants)

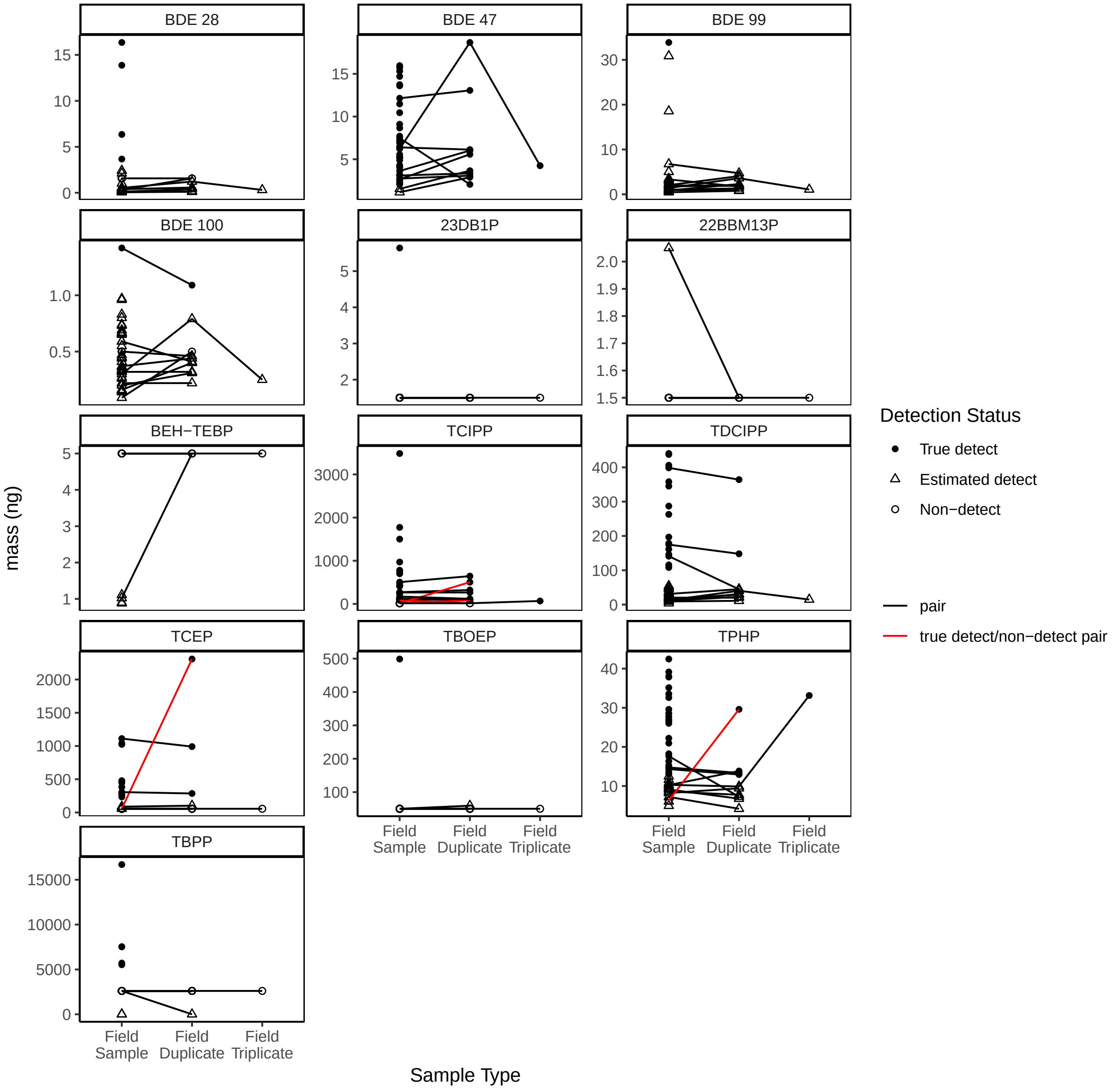

Comments:

- One sample imprecise (true detect/non-detect pair) for a few FRS – TCEP,TCIPP, and TPHP. Were there problems with this sample?
  - No clues in field log notes.
  - Surrogate recovery for BDE126, the representative surrogate for these chemicals, was low (and very similar) for both the field sample and field duplicate.
  - Both analyzed in the same batch (batch 9)

No further action indicated at this time.

Duplicates: Conclusion

- We will average duplicate pairs, setting non-detects to lab reporting limit.
- In publications, we will note the range of average RPDs across all compounds in our QA/QC discussion and note that the following compounds have average RPD $> 30\%$ (i.e. poor precision), considering duplicate pairs where both are true detects:

DEHP, *BBP*, *TCS* and *BDE 47*
